# A novel probabilistic q-rung orthopair linguistic neutrosophic information-based method for rating nanoparticles in various sectors

**DOI:** 10.1038/s41598-024-55649-7

**Published:** 2024-03-08

**Authors:** Abdul Wahab, Jawad Ali, Muhammad Bilal Riaz, Muhammad Imran Asjad, Taseer Muhammad

**Affiliations:** 1 Department of Mathematics, University of Management and Technology, Lahore, 54770 Pakistan; 2 Department of Mathematics, Quaid-e-Azam University Islamabad, Islamabad, 45320 Pakistan; 3 IT4Innovations, VSB-Technical University of Ostrava, Ostrava, Czech Republic; 4 Department of Computer Science and Mathematics, Lebanese American University, Byblos, Lebanon; 5 Department of Mathematics, College of Science, King Khalid University, Abha, Saudi Arabia

**Keywords:** Probabilistic q-rung orthopair, Linguistic, Neutrosophic set, Average and geometric aggregation operators, Nanoparticle, Decision-making, Other nanotechnology, Mathematics and computing, Nanoscience and technology, Other nanotechnology, Mathematics and computing, Nanoscience and technology

## Abstract

The idea of probabilistic q-rung orthopair linguistic neutrosophic (P-QROLN) is one of the very few reliable tools in computational intelligence. This paper explores a significant breakthrough in nanotechnology, highlighting the introduction of nanoparticles with unique properties and applications that have transformed various industries. However, the complex nature of nanomaterials makes it challenging to select the most suitable nanoparticles for specific industrial needs. In this context, this research facilitate the evaluation of different nanoparticles in industrial applications. The proposed framework harnesses the power of neutrosophic logic to handle uncertainties and imprecise information inherent in nanoparticle selection. By integrating P-QROLN with AO, a comprehensive and flexible methodology is developed for assessing and ranking nanoparticles according to their suitability for specific industrial purposes. This research contributes to the advancement of nanoparticle selection techniques, offering industries a valuable tool for enhancing their product development processes and optimizing performance while minimizing risks. The effectiveness of the proposed framework are demonstrated through a real-world case study, highlighting its potential to revolutionize nanoparticle selection in HVAC (Heating, Ventilation, and Air Conditioning) industry. Finally, this study is crucial to enhance nanoparticle selection in industries, offering a sophisticated framework probabilistic q-rung orthopair linguistic neutrosophic quantification with an aggregation operator to meet the increasing demand for precise and informed decision-making.

## Introduction

Nanoparticles, tiny particles with dimensions typically between 1 and 100 nm, are the subject of intense scientific research and technological innovation. Their small size and large surface area-to-volume ratio endow them with unique and often exceptional properties. Nanoparticles can be fashioned from a diverse array of materials, including metals, polymers, ceramics, and carbon-based substances. These minuscule entities exhibit size-dependent properties and quantum effects that distinguish them from bulk materials. As a result, they have found application in a wide range of fields. In medicine, nanoparticles are employed for targeted drug delivery, diagnostics, and novel therapies. In electronics, they feature in transistors, sensors, and quantum dots for high-resolution displays. Nanoparticles also serve as catalysts to accelerate chemical reactions, enhance energy efficiency in solar cells and batteries, and contribute to advanced materials in fields like nanocomposites and coatings. Nonetheless, the field of nanoparticles is not without challenges. Concerns about their potential toxicity to living organisms require careful consideration, and regulatory frameworks continue to evolve. Despite these challenges, nanoparticles hold great promise for the future, with ongoing research aimed at unlocking their full potential in various industries and technologies^1^. The selection of appropriate nanoparticles for various industrial applications is a critical and complex decision-making process. With the rapid growth of nanotechnology and the ever-expanding range of nanoparticles available, industries face a daunting task in choosing the right nanomaterials to meet their specific needs. To address this challenge, a novel framework has emerged, offering a systematic and informed approach to nanoparticle selection. This framework represents a pivotal shift from conventional trial-and-error methods toward a more efficient, data-driven, and risk-aware strategy for harnessing the potential of nanomaterials in industries. By integrating factors such as material properties, cost-effectiveness, environmental considerations, and safety profiles, this innovative framework aims to empower industries with the knowledge and tools necessary to make informed decisions, ultimately fostering the responsible and effective utilization of nanoparticles across diverse sectors. This introduction provides a glimpse into the evolving landscape of nanoparticle selection methodologies, setting the stage for a deeper exploration of this transformative approach. Nanofluid historical development is based on the most important contributions of many scientists and researchers. Nanofluid, a name understand by Choi and Eastman in the year 1995^2^, to describe a fluid which contain solid nanoparticles with sizes not greater than 100 nm suspended on it with solid volume fractions typically less than 4% at Argonne National Laboratory. Nano-sized particles help to improve thermal conductivity and convective heat transfer of liquids when mixed with base fluids. Xuan and Li^3^ explained that nanomaterials have increased properties (mechanical, thermal, physical, chemical), phenomena, and processes than normal materials, according to the researchers. Nanofluids got a great deal of interest as indicated by reports, the vitally main thrust for nanofluids research is an extensive variety of designing applications, including car and cooling, sunlight based and power plant cooling, transformer oil cooling, further developing diesel generator effectiveness, atomic reactor cooling, and safeguard by Wang and Mujumdar^4^. In 2007, Heris et al.^5^ arranged UV-vis range investigation that was utilized to evaluate the strength of a few nanofluids (Nanoparticle: fullerence, copper oxide, and silicon dioxide; Base liquid: DI water, ethylene glycol, and oil). They contended that the highlights of the suspended molecules and base liquids, like molecule morphology and compound construction, altogether affected the security of nanofluid. Besides, adding surfactant to the suspensions can work on their stability. In 2010, Tavman et al.^6^ investigated $\text{TiO}_{2}$, $\text{SiO}_{2}$, and $\text{Al}_{2}\text{O}_{3}$ nanoparticles in water, as well as a substantial increase in nanofluid viscosity as the nanoparticle concentration increased. In 2016, Bashirnezhad et al.^7^ examined the viscosity of nanofluids containing solid nanoparticles is higher than that of typical working fluids, and quantifying the viscosity is required for building thermal systems. The expansion in thickness is legitimate by an expansion in nanoparticle volume portion and a decline in temperature. In 2018, Shahid et al.^8^ examined the impacts of the accompanying boundaries such as, thermophoresis boundary, porousness boundary, radiation boundary etc. Determining the “best” nanoparticles depends on the specific application, goals, and requirements. Different nanofluids excel in different aspects, such as thermal conductivity enhancement, stability, compatibility, and cost-effectiveness^9,10^.

Fuzzy set theory, a cornerstone of fuzzy logic, extends classical set theory by introducing the concept of partial membership^11^. Unlike classical sets where an element either fully belongs or does not belong to a set, fuzzy sets allow for degrees of membership between 0 and 1, capturing the inherent vagueness and uncertainty in many real-world scenarios. Consider a classic example involving temperature. In classical set theory, we might define a set “Hot” for temperatures above a certain threshold, say 30 °C. In this binary framework, a temperature of 32$^{\circ }$C belongs fully to the set “Hot”, while 25 °C does not belong at all. However, using fuzzy set theory, we can represent the “Hot” set with degrees of membership. This means that a temperature of 32 °C might have a membership degree of 0.8, indicating it’s strongly “Hot”, and a temperature of 25 °C could have a membership degree of 0.3, indicating it’s somewhat “Hot.” Mathematically, fuzzy sets are defined using membership functions that assign membership degrees to elements. These functions can take various shapes, such as triangular, trapezoidal, or Gaussian, to model different degrees of uncertainty. Fuzzy set theory finds applications in various fields, including control systems, decision-making, pattern recognition, and artificial intelligence. It allows for a more nuanced representation of linguistic terms and provides a framework for handling imprecise or vague information that arises in many real-world scenarios.

In real world circumstances, due to the growing ambiguities and uncertainties, the issues of decision-making^12^ have gotten increasingly complicated recently across a variety of sectors. Researchers have proposed a variety of fuzzy set types^13^, intuitionistic fuzzy^14^, hesitant fuzzy^15^, and linguistic variables^16^, to describe and express the confusing and vague information during making decisions situations in order to address these obstacles. A mathematical framework called fuzzy set theory was developed to cope with ambiguity and uncertainty in data. An element can belong to a set with a degree of membership between 0 and 1, rather than a binary value of 0 or 1, in this expansion of traditional set theory. Control systems, artificial intelligence, decision-making, and pattern identification have all benefited from the use of fuzzy sets^17^. Fuzzy set theory has been extended to solve the drawbacks of conventional fuzzy sets with the Q-Rung fuzzy set^18^. A generalized orthopair fuzzy set (GOFS), also known as a q-rung orthopair fuzzy set (q-ROFS), is a higher variant of ordinary fuzzy sets by relaxing restrictions on the degrees of membership and non-membership^19^. A Q-rung orthopair fuzzy set (QROFS) is a mathematical concept that extends the traditional notion of fuzzy sets to accommodate more nuanced and refined representations of uncertainty and ambiguity. Introduced as an enhancement to the existing orthopair fuzzy set framework, QROFS incorporates the concept of a Q-rung orthopair, where the degree of membership and non-membership of an element in a set is expressed using two distinct membership functions^20^. These functions capture the uncertainty in a more granular manner, providing a more flexible and adaptable model for dealing with complex decision-making scenarios. The QROFS finds applications in various domains, particularly in decision support systems, pattern recognition, and artificial intelligence^21^. The q-Rung orthopair fuzzy set is more likely to find usage in decision science since it can easily address ambiguous issues that are outside the purview of the previously described generalized fuzzy sets. One crucial information measure for determining various criteria in decision-making situations is the idea of composite relations^22^. In it, the idea of q-rung linguistic variables is introduced. These variables are used to characterise the degree to which an element belongs to a set based on linguistic words. The linguistic variables of q-rung offer a more adaptable and precise representation of the ambiguity and uncertainty in data. A generalisation of fuzzy set theory called Q-rung fuzzy set theory^23^ enables the representation of ambiguous and imprecise information in decision-making procedures. A q-rung linguistic variable in Q-rung fuzzy sets represents the level of membership of an element in the set. The q-rung linguistic variable, which has a maximum value of r and a range of 0 to r, determines an element’s level of membership based on a linguistic term. The Q-rung fuzzy set theory has been used in a variety of areas, including pattern detection, decision-making, and image processing. Another addition to classical set theory that enables the representation of ambiguous or partial information is neutrosophic set theory^24,25^. The truthness degree, indeterminacy degree, and falsity degree are the three factors that neutrosophic sets utilise to describe an element’s membership in a set. The amount to which an element is true is indicated by its degree of truth, and the extent to which an element is false is shown by its degree of falsity. The degree of indeterminacy is a measure of how uncertain an element’s truth or falsehood^26^. Numerous disciplines, including artificial intelligence, decision-making, and image processing, have used neutrosophic set theory. Combining q-rung fuzzy set theory with neutrosophic set theory, linguistic neutrosophic fuzzy set theory^27^ enables the representation and manipulation of ambiguous, incomplete, and hazy information in decision-making processes. Decision-making, pattern recognition, and image processing are just a few of the areas where linguistic neutrosophic fuzzy set theory has been used^28^. However, these fuzzy sets have difficulties when it comes to handling the ambiguities and uncertainties that emerge during decision-making processes, particularly when the linguistic variables are utilised to convey the decision-makers’ viewpoints^29^. The idea of neutrosophic sets, an extension of fuzzy sets that can manage the ambiguity, inconsistently^30^, and incompleteness in decision-making scenarios, was presented to solve these shortcomings. Neutosophic sets, however, also have limits when it comes to handling difficult decision-making circumstances including language factors. As an extension of neutrosophic sets and linguistic variables, q-rung linguistic neutrosophic fuzzy (QRLNF) sets were proposed to get over these restrictions^31^. The weight is utilised to indicate the weight of the alternative in the decision-making process, whereas the q-rung linguistic phrase represents the degrees of membership of the linguistic variable. The notions of q-rung linguistic variables and neutrosophic sets are combined to form neutrosophic (q-LNS) fuzzy sets^32^.

A probabilistic, linguistic term, a degree of membership, and a degree of non-membership are all included in q-rung linguistic words, which are used to represent the membership degrees of elements in P-q-LNS fuzzy sets^33^. The degree of non-membership describes the degree to which an element does not belong to the linguistic term, whereas the degree of membership indicates the truth value of an element belonging to a certain linguistic word. Complex decision-making issues may be handled with the P-q-LNS fuzzy sets, especially when working with shaky and ambiguous data. They give decision-making processes a more thorough framework for describing and managing ambiguity, vagueness, and indeterminacy^34^. Decision-makers in a variety of industries, including banking, medical, engineering, and decision support systems, can manage more complex and ambiguous information by employing q-LNS fuzzy sets, which enables them to make more educated judgements^35^. QLNF operators, such as QLNF weighted aggregation^36^ operators and QLNF hesitant fuzzy linguistic term sets, to perform various decision-making tasks in a Q-LNs framework. Q-RLNs has been applied in various fields, including finance, engineering, medicine, and environmental science, to address the challenges of decision-making in uncertain and complex systems. The QLNF approach has also been used in ranking assessment, where it has been shown to outperform traditional ranking methods^37^. In this regard, suggests a novel method for solving decision-making issues in which the alternatives and criteria are assessed in a Q-RLNs environment. The authors introduce several Q-RLNs weighted aggregation operators. The properties and characteristics of these operators are analyzed, and their performance is compared with other existing aggregation operators. Finally, the proposed approach is illustrated through a real-world case study of rankings of a different nanoparticles in different industries. The results demonstrate the effectiveness and applicability of the suggested method for resolving decision-making issues using P-QRLNs data. Overall, this research provides a valuable contribution to the field of decision making by introducing a new approach that can handle complex decision making situations involving P-QRLNs information.

The main objective of this work is to present a thorough technique that systematically takes into consideration the many assessments and inherent uncertainties related to nanoparticle ranks. The method aims to provide a consensus ranking that accurately reflects the complex and thorough assessment of nanofluids by balancing various information sources and expert opinions. By using Neutrosophic weighted aggregation operations with probabilistic q-rung linguistics, the goal is to effectively handle the uncertainties and complexity included in nanoparticle ranks. This entails combining disparate language viewpoints and weighted preferences in order to finally provide a more accurate, dependable, and thorough evaluation of nanoparticle performance and overall rankings in the fluid dynamics domain.

In this paper our contributions are as follows:

- The P-QRLNs-based framework is a novel method for rating nanoparticles in various sectors that offers a more thorough and precise assessment.
- The suggested P-QRLNs-based technique outperforms conventional approaches thanks to its improved ranking of various nanoparticles accuracy, consistency, and dependability.
- Implementing the suggested technique has the potential to significantly improve the ranking of nanoparticles’ thermal conductivity and stability while providing smooth ranking experiences.
- We provide a hybrid degree of probabilistic and linguistic with Q-rung orhopair Neutrosopic to get a generic structure called P-QRLNs to deal with the issue of uncertainty with positive, indeterminacy, and negative membership degrees.

The motivation for a research paper is rooted in the growing significance of nanotechnology in industrial applications. The growing importance of nanotechnology across several industries is driving this study. Interest in industries including healthcare, electronics, energy, and manufacturing has increased due to the extraordinary qualities of nanoparticles. However, enterprises hoping to properly use the promise of nanoparticles face significant challenges due to their complex structure and wide diversity. Critical elements such as the spread of nanoparticles, the difficulty of making decisions, the need to reduce risk, the need for economy and efficiency, and the introduction of new language and neutroscientific instruments serve as the foundation for this study’s motivation. The urgent need to close the knowledge gap between the vast array of nanoparticles accessible and their strategic application in industry is the focus of this research. By presenting a novel framework that combines sophisticated mathematical and linguistic methods, the study seeks to provide industry with a thorough, data-driven, and risk-aware approach. By using this technique, nanoparticles may be carefully chosen, maximizing their potential and pushing the boundaries of industrial application technology.

The importance of this new framework introduces a sophisticated method for choosing nanoparticles, which is of great importance to enterprises. The system tackles the intricacies involved in nanoparticle assessment by combining probabilistic q-rung orthopair linguistic neutrosophic quantification with an effective aggregation operator. This novel approach improves the accuracy of decision-making when choosing a variety of nanoparticles, which benefits industrial operations by increasing performance, sustainability, and efficiency. The framework offers a simplified method and a deep knowledge for selecting the best nanoparticles for a variety of applications, making it an invaluable tool for industry experts.

Lastly the structure of the paper is as follows: Basic preliminaries are discussed in “Preliminaries” section while a unique framework is developed in “Basic operations” section. The “Probabilistic q-ROLNS aggregation operators” section explores a few aggregation operators, including P-QRLNs averaging operators like arithmetic and geometric, and their fundamental properties. P-QRLNs and their basic operations. In “An innovative technique for effective decision-making” section, we provide a methodical procedure for dealing with problem-solving situations based on probabilistic q-rung linguistic neutrosophic operators. For purposes of application, in “Explanatory example” section we take into account a nanaoparticles ranking based on the probabilistic q-rung linguistic neutrosophic. We compute the sensitivity analysis for various values of “q” in “Managerial and policy implications for proposed technique” section and provide graphs for both arithmetic and geometric data. To prove the new model’s superiority, we compare it to the current model in “Sensitivity analysis” section of our paper. Give a conclusion and future work in “Conclusions and future initiatives” section.

## Preliminaries

In this section, the concepts of Probabilistic q-RLNs and its historical context will be provided. We will define some terms such as fuzzy set and characteristic of fuzzy set, q-rung orthopair fuzzy set, Neutrosophic set and its properties etc.

To deal with the ambiguous nature of data, fuzzy sets were initially devised in 1965 by Zadeh^38^.

### Definition 1

(*Fuzzy set*)^39^ The concept of fuzzy-set $\xi$ defined as $\xi = \{( f'', F_\xi ( f''))|f'' {\displaystyle \in }N'\}$ such that $F_\xi : N' {\displaystyle \rightarrow } I$, where $F_\xi ( f'')$ denotes the belonging value of $f'' {\displaystyle \in } \xi .$

### Definition 2

(*Characteristic of fuzzy sets*)^40^ Let us suppose that there are two fuzzy-sets say $\xi$ and $\rho$ then $\forall$ $f'' {\displaystyle \in } N'$, then

i. $$\xi {\displaystyle \cup }\rho = \{( f'', max\{F_\xi (f''), F_{\rho }( f'')\})\}.$$
ii. $$\xi {\displaystyle \cap } \rho = \{( f'', min\{F_\xi (f''), F_{\rho }( f'')\})\}.$$
iii. $$\xi ^f = \{(f'', 1 - F_\xi ( f''))|f'' {\displaystyle \in } N'\}.$$

### Definition 3

(*q-Rung orthopair fuzzy set*)^41^ Assume that the set of universal discourse is represented by $\digamma$, a q-ROFS on $\digamma$ is defined in below:

$$\begin{aligned} B'_{1} =(\ddot{m},\{\Theta _{B'_{1}}(\ddot{m}),\Pi _{B'_{1}}(\ddot{m})\}|\ddot{m}\in \digamma ), \end{aligned}$$

$\Theta _{B'_{1}}(\ddot{m})$ in closed unit interval is referred as degree of membership of $B'_{1}$, $\Pi _{B'_{1}}(\ddot{m})$ is referred as degree of non-membership of $B'_{1}$ and $\Theta _{B'_{1}}(\ddot{m})$, $\Pi _{B'_{1}}(\ddot{m})$ holds the relation: $0\le \Theta _{B'_{1}}(\ddot{m})^{q}+\Pi _{B'_{1}}(\ddot{m})^{q}\le 1$ for $\forall \ddot{m} \in B'_{1}$. Then indeterminacy degree of $\ddot{m}$ in $B'_{1}$ is denoted by $\mathbf {\pi }_{B'_{1}}(\ddot{m})=(\Theta _{B'_{1}}(\ddot{m})^{q}+\Pi _{B'_{1}}(\ddot{m})^{q}-\Theta _{B'_{1}}(\ddot{m})^{q}\Pi _{B'_{1}}(\ddot{m})^{q})^{1/q}$^42^.

### Definition 4

(*Neutrosophic set*)^43,44^ A neutrosophic set (NS) defined on $\digamma$ is explained here:

$$\begin{aligned} B_{2}=(\ddot{m},\{\Theta _{B_{2}}(\ddot{m}),\Psi _{B_{2}}(\ddot{m}),\Pi _{B_{2}}(\ddot{m})\}|\ddot{m}\in \digamma ), \end{aligned}$$

$\Theta _{B_{2}}(\ddot{m})\in$ [0, 1] is referred as truth membership degree of $B_{2}$, $\Psi _{B_{2}}(\ddot{m})\in$ [0, 1] is addressed by neutral-membership degree of $B_{2}$ and $\Pi _{B_{2}}(\ddot{m})\in$ [0, 3] is referred as false membership degree of $B_{2}$ and $\Theta _{B_{2}}(\ddot{m})$,$\Psi _{B_{2}}(\ddot{m})$,$\Pi _{B_{2}}(\ddot{m})$ holds the following condition: $0\le [\Theta _{B_{2}}(\ddot{m})+\Psi _{B_{2}}(\ddot{m})+\Pi _{B_{2}}(\ddot{m})]\le 3$ for $\forall \ddot{m} \in B_{2}$. Then $\pi _{B_{2}}(\ddot{m})=1-\Theta _{B_{2}}(\ddot{m})-\Pi _{B_{2}}(\ddot{m})$ is termed as refusal-membership degree of $\ddot{m}$ in $B_{2}$. The refusal membership degree signifies the uncertainty or indecision associated with the inclusion or exclusion of an element in the set.

### Definition 5

(*Property of neutrosophic set*)^45^ The NS $\grave{T}$ is present in another NS $\grave{L}$, showed by $\grave{T}\subseteq \grave{L}$, if and only if $inf T\grave{T}(\ddot{m})\le inf T\grave{L}(\ddot{m})$, $sup T\grave{T}(\ddot{m})\le sup T\grave{T}(\ddot{m})$, $inf I\grave{T}(\ddot{m})\ge inf I\grave{L}(\ddot{m})$, $sup I\grave{T}(\ddot{m})\ge sup I\grave{L}(\ddot{m})$, $inf F\grave{T}(\ddot{m})\ge inf F\grave{L}(\ddot{m})$ and $sup F\grave{T}(\ddot{m})\ge sup F\grave{L}(\ddot{m})$ for any $\ddot{m}\in \digamma$.

### Definition 6

(*q-rung orthopair neutrosophic set*)^46^ A q-rung neutrosophic set (q-RNS) on $\digamma$ is explained in below:

$$\begin{aligned} B_{3}=(\ddot{m},\{\Theta _{B_{3}}(\ddot{m}),\Psi _{B_{3}}(\ddot{m}),\Pi _{B_{3}}(\ddot{m})\}|\ddot{m}\in \digamma ), \end{aligned}$$

satisfy the following given condition: $0\le [\Theta _{B_{3}}(\ddot{m})^{q}+\Psi _{B_{3}}(\ddot{m})^{q}+\Pi _{B_{3}}(\ddot{m})^{q}]\le 3$ for $\forall \ddot{m} \in B_{3}$. Then $\pi _{B_{3}}(\ddot{m})=(1-(\Theta _{B_{3}}(\ddot{m}))^{q}+(\Psi _{B_{3}}(\ddot{m}))^{q}+(\Pi _{B_{3}}(\ddot{m}))^{q})^{1/q}$ is called to as the refusal-membership degree $\ddot{m}$ in $B_{3}$.

### Definition 7

^47^ Suppose that if we have $\varepsilon$ = [0,1], then the given this mapping $\breve{\digamma }$ : $\varepsilon \times \varepsilon \rightarrow \varepsilon$ known as $(t-Norm)$ if for $a_{0}, a_{1}, a_{2}\in \varepsilon,$

1. $\breve{\digamma }$ is monotonic, associative and also continuous.
2. $$\breve{\digamma }$$(a,1) = a.

### Definition 8

^48^ Suppose that if we have $\varepsilon$ = [0,1], then the given this mapping $\breve{\digamma }$ : $\varepsilon \times \varepsilon \rightarrow \varepsilon$ known as $(t-Conorm)$ if for $a_{0}, a_{1}, a_{2}\in \varepsilon$,

1. $\breve{\digamma }$ is monotonic, associative and also continuous.
2. $$\breve{\digamma }$$(a,0) = a.

### Definition 9

^49^ A t-Norm $\ddot{T}$ to be known as the Archimedean triangular-norm, the following properties must hold:

1. It is continuous.
2. $$\ddot{T}({\check{a}}$$,$${\check{a}})< {\check{a}}$$ $$\forall {\check{a}} \in$$ (0,1).

### Definition 10

^50^ A t-Conorm $\breve{\digamma }$ to be known as the Archimedean triangular-Conorm, the following properties must hold:

1. It is continuous.
2. $$\breve{\digamma }({\check{a}}$$,$${\check{a}})> {\check{a}}$$ $$\forall {\check{a}} \in$$ (0,1).

## Basic operations

### Definition 11

(*probabilistic q-rung orthopair linguistic neutrosophic set*) Let us suppose that a probabilistic q-rung orthopair linguistic neutrosophic set (P-QRLNS) *L* on $\digamma$ is explained in below:

1 $$\begin{aligned} B_{4}=\frac{p_{i}}{\{(s'_{\alpha }(\ddot{m}),\{\Theta _{A_{4}}(\ddot{m}),\Psi _{A_{4}}(\ddot{m}),\Pi _{A_{4}}(\ddot{m})\}|\ddot{m}\in \digamma )\}} \,\,\,\ and\,\,\,\,\,\ (i=1,2,3,...,\Bbbk ). \end{aligned}$$

where, $s_{\alpha }(\ddot{m})\in \textbf{S}$, *p* is the probabilistic degree and satisfy the given condition: $0\le \Theta _{B_{4}}(\ddot{m})^{q}+\Psi _{B_{4}}(\ddot{m})^{q}+\Pi _{B_{4}}(\ddot{m})^{q}\le 3$ for $\forall \ddot{m} \in \digamma$. Then $\pi _{B_{4}}(\ddot{m})=(1-(\Theta _{B_{4}}(\ddot{m}))^{q}+(\Psi _{B_{4}}(\ddot{m}))^{q}+(\Pi _{B_{4}}(\ddot{m}))^{q})^{1/q}$ is referred as refusal-membership degree of $\ddot{m}$ in $B_{4}$.

For the purpose of simplicity, $(s_{\alpha }(\ddot{m})$,$\{\Theta _{B_{4}}(\ddot{m})$,$\Psi _{B_{4}}(\ddot{m})$,$\Pi _{B_{4}}(\ddot{m})$}) is referred as (q-RLNN) which is denoted as $\varsigma =(s_{\alpha }$,$\{\Theta$,$\Psi$,$\Pi \})$.

The below score function and accuracy function are the modified version of^51,52^.

### Definition 12

(*Score function*) Suppose that $\varsigma =\frac{p}{(s_{\alpha },\{\Theta ,\Psi ,\Pi \})}$ is a P-QRLNN, then the term Score-function is,

2 $$\begin{aligned} Scr(\varsigma )= \alpha \times (\Theta ^{q}+1-\Pi ^{q})^{p}. \end{aligned}$$

### Definition 13

(*Accuracy function*) Suppose that $\varsigma =\frac{p}{(s_{\alpha },\{\Theta ,\Psi ,\Pi \})}$ is a P-QRLNN, then the term Accuracy-function is,

$$\begin{aligned} H(\ddot{\psi })= \alpha \times (\Theta ^{q}+\Psi ^{q}+\Pi ^{q})^{p}. \end{aligned}$$

### Definition 14

^53^ Suppose that $\ddot{\eth }_{1}=\frac{p}{(s_{\alpha _{1}}, \{\Theta _{1}, \Psi _{1}, \Pi _{1}\})}$ and $\ddot{\eth }_{2}=\frac{p'}{(s_{\alpha _{2}}, \{\Theta _{2}, \Psi _{2}, \Pi _{2}\})}$ be any two P-QRLNN, Scr($\ddot{\eth }_{1}$) and Scr($\ddot{\eth }_{2}$) is a Score-function of $\ddot{\eth }_{1}$ and $\ddot{\eth }_{2}$, H($\ddot{\eth }_{1}$) and H($\ddot{\eth }_{2}$) is an Accuracy-function of $\ddot{\eth }_{1}$ and $\ddot{\eth }_{2}$.

1. Suppose that if we have Scr($\ddot{\eth }_{1})> Scr(\ddot{\eth }_{2})$, which implies that $\ddot{\eth }_{1}>\ddot{\eth }_{2}.$
2. Suppose that if we have Scr($\ddot{\eth }_{1})= Scr(\ddot{\eth }_{2})$, which implies that $\ddot{\eth }_{1}=\ddot{\eth }_{2}.$
3. Suppose that if we have H($\ddot{\eth }_{1})> H(\ddot{\eth }_{2})$, which implies that $\ddot{\eth }_{1}>\ddot{\eth }_{2}.$
4. Suppose that if we have H($\ddot{\eth }_{1})= H(\ddot{\eth }_{2})$, which implies that $\ddot{\eth }_{1}=\ddot{\eth }_{2}.$

Based on the t-conorm and t-norm of Archimedian^54^, The previous q-rung linguistic picture fuzzy operational rules are expanded to a more generic form in this section. For this, a bijective-function $g: [a,b] \subseteq {\mathbb {R}}$ in unit interval defined as $g(t)=\frac{t-a_{1}}{t-a_{2}}, \forall t \in [a_{1},a_{2}]$, is utilized. Letting $a_{1}=0, a_{2}=\ddot{\xi }$, then $g:[0,\ddot{\xi }]$ in unit interval.

### Definition 15

Suppose that $\Delta =\{\frac{p}{(\acute{s}_{\alpha }, \{\Theta ,\Psi ,\Pi \})}\}$, $\Delta _{1}=\{\frac{p'}{(\acute{s}_{\alpha _{1}}, \{\Theta _{1}, \Psi _{1} , \Pi _{1}\})}\}$ and $\Delta _{2}=\{\frac{p''}{(\acute{s}_{\alpha _{2}}, \{\Theta _{2}, \Psi _{2}, \Pi _{2}\})}\}$ be any three P-QRLNS and $\Delta$ be positive real number, then the following operations are follow as:

1. Additive operation:
  $$\begin{aligned} \Delta _{1}\oplus \Delta _{2}= & {} \bigg (\acute{s}_{\acute{g}^{-1}(\varkappa ^{-1}(\varkappa (\acute{g}(\alpha _{1}))+\varkappa (\acute{g}(\alpha _{2})))},\\ {}{} & {} \bigg \{{(\varkappa ^{-1}(\varkappa (\Theta _{1}^{q}) +\varkappa (\Theta _{2}^{q}))}^{p/q},(\Pi ^{-1}(\Pi (\Psi _{1})+\Pi (\Psi _{2}))^{p'},(\Pi ^{-1}(\Pi (\Pi _{1})+\Pi (\Pi _{2}))^{p'}\bigg \}\bigg ); \end{aligned}$$
2. Multiplication:
  $$\begin{aligned} \Delta _{1}\otimes \Delta _{2}= & {} \bigg (\acute{s}_{\acute{g}^{-1}(\Pi ^{-1}(\Pi (\acute{g}(\alpha _{1}))+\Pi (\acute{g}(\alpha _{2}))))},\\{} & {} \bigg \{{(\Pi ^{-1}(\Pi (\Theta _{1})+\Pi (\Theta _{2}))))^{p'},(\Pi ^{-1}(\Pi (\Psi _{1})+\Pi (\Psi _{2})))^{p'},(\varkappa ^{-1}(\varkappa (\Pi _{1}^{q})+(\varkappa (\Pi _{2}^{q}))}^{p/q}\ \bigg \}\bigg ); \end{aligned}$$
3. Scalar-multiplication:
  $$\begin{aligned} \gimel \Delta= & {} \bigg (\acute{s}_{\acute{g}^{-1}(\varkappa ^{-1}(\gimel \varkappa (\acute{g}(\alpha ))))},\\ {}{} & {} \bigg \{(\varkappa ^{-1}(\gimel \varkappa (\Theta ^{q})))^{p/q},(\Pi ^{-1}(\gimel \Pi (\Psi ))),(\Pi ^{-1}(\gimel \Pi (\Pi )))^{p'}\bigg \}\bigg ); \end{aligned}$$
4. Power operation:
  $$\begin{aligned} \Delta ^{\gimel }= & {} \bigg (\acute{s}_{\acute{g}^{-1}(\Pi ^{-1}(\gimel \Pi (\acute{g}(\alpha ))))},\\{} & {} \bigg \{(\Pi ^{-1}(\gimel \Pi (\Theta ))^{p},(\Pi ^{-1}(\gimel \Pi (\Psi )))^{p'},(\varkappa ^{-1}(\gimel \varkappa (\Pi ^{q})))^{p/q}\bigg \}\bigg ). \end{aligned}$$

### Theorem 1

Suppose that $\Delta =\frac{p}{(\acute{s}_{\alpha }, \{\Theta ,\Psi ,\Pi \})}$, $\Delta _{1}=\frac{p'}{(\acute{s}_{\alpha _{1}}, \{\Theta _{1}, \Psi _{1} , \Pi _{1}\})}$ and $\Delta _{2}=\frac{p''}{(\acute{s}_{\alpha _{2}}, \{\Theta _{2}, \Psi _{2}, \Pi _{2}\})}$ be any three P-QRLNNs and $\gimel _{1},\gimel _{2},\gimel _{3}\ge {0}$, then the following rules must be holds for these three scalars.

1. $$\Delta _{1}\oplus \Delta _{2} = \Delta _{2}\oplus \Delta _{1}.$$
2. $$\Delta _{1}\otimes \Delta _{2} =\Delta _{2}\otimes \Delta _{1}.$$
3. $$\gimel \odot (\Delta _{1}\oplus \Delta _{2}) = (\gimel \odot \Delta _{2})\oplus ( \gimel \odot \Delta _{1}).$$
4. $$(\Delta _{1}\otimes \Delta _{2})^{\gimel } = (\Delta _{2})^{\gimel }\otimes (\Delta _{1})^{\gimel }.$$
5. $$(\gimel _{1}\odot \Delta )\oplus (\gimel _{2}\odot \Delta ) = (\gimel _{1}+\gimel _{2})\odot \Delta .$$
6. $$(\Delta )^{\gimel _{1}}\otimes (\Delta )^{\gimel _{2}} = (\Delta )^{(\gimel _{1}+\gimel _{2})}.$$

### Theorem 2

Let us suppose that $\Delta _{0} =\frac{p}{(\acute{s}_{\alpha }, \{\Theta ,\Psi ,\Pi \})}$, $\Delta _{1}=\frac{p'}{(\acute{s}_{\alpha _{1}}, \{\Theta _{1}, \Psi _{1} , \Pi _{1}\})}$ and $\Delta _{2}=\frac{p''}{(\acute{s}_{\alpha _{2}}, \{\Theta _{2}, \Psi _{2}, \Pi _{2}\})}$ be any three P-QRLNS and $\gimel$, thus, for addition and multiplication, the associative rules are:

1. $$(\Delta _{0}\oplus \Delta _{1})\oplus \Delta _{2} = \Delta _{1}\oplus (\Delta _{0}\oplus \Delta _{2}).$$
2. $$\Delta _{0}\otimes (\Delta _{1}\otimes \Delta _{2}) = (\Delta _{1}\otimes \Delta _{0})\otimes \Delta _{3}.$$

## Probabilistic q-ROLNS aggregation operators

We investigate the probabilistic q-Rung orthopair linguistic neutrosophic operators according to defined average arithmetic and geometric operations in this section.

###  Probabilistic q-Rung orthopair linguistic neutrosophic number weighted averaging aggregation operator

#### Definition 16

^55^ Suppose we say $\ddot{\delta }_{\iota }=\frac{p_{i}}{(\acute{s}_{\beta _{\iota }} ,\{\Theta _{\iota },\varepsilon _{\iota },\Pi _{\iota }\})}$, $(\iota =1,2,3,...,\Bbbk )$ and $(i=1,2,3,...,\Bbbk )$ are the P-QRLNs, the based upon Archimedean t-conorm and t-norm, the operator P-QRLNWAA defined as follow:

$$\begin{aligned} P{\text{-}}QRLNWAA(\ddot{\delta }_{1},\ddot{\delta }_{2},\ddot{\delta }_{3},...,\ddot{\delta }_{\Bbbk })= & {} \overset{\Bbbk }{\underset{\iota =1}{\oplus }} (\gamma _{\iota }\odot \ddot{\delta }_{\iota }), \end{aligned}$$

where $\gamma _{\iota }=(\gamma _{1},\gamma _{2},\gamma _{3},...,\gamma _{\Bbbk })^{T}$ are weighted vectors, such that $\gamma _{\iota }\in [0,1]$ and $\overset{\Bbbk }{\underset{\iota =1}{\sum }}\gamma _{\iota }= 1.$ Similarly, $p_{i}\in [0,1]$ and $\overset{\Bbbk }{\underset{i=1}{\sum }}p_{i}= 1.$

#### Theorem 3

Let us suppose that $\ddot{\delta }_{\iota }=\frac{p_{i}}{(\acute{s}_{\beta _{\iota }} ,\{\Theta _{\iota },\varepsilon _{\iota },\Pi _{\iota }\})}$, $(\iota =1,2,3,...,\Bbbk )$ and $(i=1,2,3,...,\Bbbk )$ be an array of P-QRLNs. The P-QRLNWAA is defined as,

$$\begin{aligned} P{\text{-}}q{\text{-}}RLNWAA(\ddot{\delta }_{1},\ddot{\delta }_{2},\ddot{\delta }_{3},...,\ddot{\delta }_{\Bbbk })= & {} \left( \acute{s}_{\acute{g}^{-1}(\Psi ^{-1}(\overset{\Bbbk }{\underset{\iota = 1}{\sum }}\gamma _{\iota }\Psi (\acute{g}(\beta _{\Bbbk }))))},\right. \\{} & {} \left\{ \left( \Psi ^{-1}\left( \overset{\Bbbk }{\underset{\iota = 1}{\sum }} \gamma _{\iota }\Psi (\Theta _{\iota }^{q})\right) \right) ^{p_{i}/q},\left( \Pi ^{-1}\left( \overset{\Bbbk }{\underset{\iota = 1}{\sum }}\gamma _{\Bbbk }\Pi (\varepsilon _{\Bbbk })\right) \right) ^{p_{i}},\right. \\{} & {} \quad \left. \left. \left( \Pi ^{-1}\left( \overset{\Bbbk }{\underset{\iota = 1}{\sum }}\gamma _{\Bbbk }\Pi (\Pi _{\Bbbk })\right) \right) ^{p_{i}}\right\} \right) . \end{aligned}$$

#### *Proof*

We demonstrate utilising the Mathematical-induction approach.

- Here, take $\Bbbk =2$, then
  $$\begin{aligned}{} & {} P{\text{-}}QRLNWAA(\ddot{\delta }_{1},\ddot{\delta }_{2}) = \gamma _{1} \ddot{\delta }_{1}\oplus \gamma _{2} \ddot{\delta }_{2} \\{} & {} \quad = \bigg (\acute{s}_{\acute{g}^{-1}(\Psi ^{-1}(\gamma _{1}\Psi (\acute{g}(\beta _{1}))))},\bigg \{(\Psi ^{-1}(\gamma _{1}\Psi (\Theta _{1}^{q})))^{p_{i}/q}, (\Pi ^{-1}(\gamma _{1} \Pi (\varepsilon _{1})))^{p_{i}},(\Pi ^{-1}(\gamma _{1} \Pi (\Pi _{1})))^{p_{i}}\bigg \}\bigg )\oplus \\{} & {} \qquad \bigg (\acute{s}_{\acute{g}^{-1}(\Psi ^{-1}(\gamma _{2}\Psi (\acute{g}(\beta _{2}))))},\bigg \{(\Psi ^{-1}(\gamma _{2} \Psi (\Theta _{2}^{q})))^{p_{i}/q},(\Pi ^{-1}(\gamma _{2} \Pi (\varepsilon _{2})))^{p_{i}},(\Pi ^{-1}(\gamma _{2} \Pi (\Pi _{2})))^{p_{i}}\bigg \}\bigg )\\{} & {} \quad = \bigg (\acute{s}_{\acute{g}^{-1}(\Psi ^{-1}(\Psi (\acute{g}(\acute{g}^{-1}(\Psi ^{-1}(\gamma _{1}\Psi (\acute{g}(\beta _{1})))))) +\Psi (\acute{g}(\acute{g}^{-1}(\Psi ^{-1}(\gamma _{2}\phi (\acute{g}(\beta _{2}))))))))},\\{} & {} \qquad \bigg \{(\Psi ^{-1}(\Psi (\Psi ^{-1}(\gamma _{1} \phi (\Theta _{1}^{q})))^{p_{i}/q})^{q}+\Psi ((\phi ^{-1}(\gamma _{2} \phi (\Theta _{2}^{q})))^{1/q})^{q})^{{p_{i}}/q},(\Pi ^{-1}(\Pi (\Pi ^{-1}(\gamma _{1} \Pi (\varepsilon _{1})))\\{} & {} \qquad +\Pi ((\Pi ^{-1}(\gamma _{2} \Pi (\varepsilon _{1}))))))^{p_{i}},\\{} & {} \qquad (\Pi ^{-1}(\Pi ((\Pi ^{-1}(\gamma _{1} \Pi (\Pi _{1})))+\Pi ((\Pi ^{-1}(\gamma _{2} \Pi (\Pi _{2})))))))^{p_{i}}\bigg \}\bigg ) \\{} & {} \quad = \left( \acute{s}_{\acute{g}^{-1}(\Psi ^{-1}(\overset{2}{\underset{\Bbbk =1}{\sum }}\gamma _{\Bbbk }\Psi (\acute{g}(\beta _{\iota }))))^{p_{i}}},\right. \\{} & {} \qquad \left. \left\{ \left( \Psi ^{-1}\left( \overset{2}{\underset{\iota =1}{\sum }} \gamma _{\iota }\Psi (\Theta _{\iota }^{q})\right) \right) ^{^{p_{i}}/q},\left( \Pi ^{-1}\left( \overset{2}{\underset{\iota =1}{\sum }}\gamma _{\iota }\Pi (\varepsilon _{\iota })\right) \right) ^{p_{i}}, \left( \Pi ^{-1}\left( \overset{2}{\underset{\iota =1}{\sum }}\gamma _{\iota }\Pi (\Pi _{\iota })\right) \right) ^{p_{i}}\right\} \right) . \end{aligned}$$
- We assume that any value of $\Bbbk =\acute{t}$ for which our result holds true,
  $$\begin{aligned}{} & {} P{\text{-}}QRLNWAA(\ddot{\delta }_{1},\ddot{\delta }_{2},\ddot{\delta }_{3},...,\ddot{\delta }_{\acute{t}}) = \left( \acute{s}_{\acute{g}^{-1}\left( \Psi ^{-1}\left( \overset{\acute{t}}{\underset{\iota =1}{\sum }}\gamma _{\iota }\Psi (\acute{g}(\beta _{\iota }))\right) \right) },\right. \\{} & {} \quad \left. \left\{ \left( \Psi ^{-1}\left( \overset{\acute{t}}{\underset{\iota =1}{\sum }} \gamma _{\iota }\Psi (\Theta _{\iota }^{q})\right) \right) ^{{p_{i}}/q},\left( \Pi ^{-1}\left( \overset{\acute{t}}{\underset{\iota =1}{\sum }}\gamma _{\iota }\Pi (\varepsilon _{l})\right) \right) ^{p_{i}}, \left( \Pi ^{-1}\left( \overset{\acute{t}}{\underset{\iota =1}{\sum }}\gamma _{\iota }\Pi (\Pi _{\iota })\right) \right) ^{p_{i}}\right\} \right) . \end{aligned}$$
- By utilising (a) and (b) in this section, we can now demonstrate that our solution holds true for any $\Bbbk =\acute{t}+1$.
  $$\begin{aligned}{} & {} P{\text{-}}QRLNWAA(\ddot{\delta }_{1},\ddot{\delta }_{2},\ddot{\delta }_{3},...,\ddot{\delta }_{\acute{t}},\ddot{\delta }_{\acute{t}+1})= \overset{\acute{t}}{\underset{\iota =1}{\oplus }} (\gamma _{\iota }\odot \ddot{\delta }_{\iota })\oplus (\gamma _{acute{t}+1}\odot \ddot{\delta }_{\acute{t}+1}) \\{} & {} \quad = \left( \acute{s}_{\acute{g}^{-1}\left( \Psi ^{-1}\left( \overset{\Bbbk }{\underset{\iota = 1}{\sum }}\gamma _{\iota }\Psi (\acute{g}(\beta _{\iota }))\right) \right) },\right. \\{} & {} \qquad \left. \left\{ \left( \Psi ^{-1}\left( \overset{\Bbbk }{\underset{\iota =1}{\sum }} \gamma _{\iota }\Psi (\Theta _{\iota }^{q})\right) \right) ^{{p_{i}}/q},\left( \Pi ^{-1}\left( \overset{\Bbbk }{\underset{\iota =1}{\sum }}\gamma _{\iota }\Pi (\varepsilon _{\iota })\right) \right) ^{p_{i}}, \left( \Pi ^{-1}\left( \overset{\Bbbk }{\underset{\iota = 1}{\sum }}\gamma _{\iota }\Pi (\Pi _{\iota })\right) \right) ^{p_{i}}\right\} \right) ,\\{} & {} \quad = \left( \acute{s}_{\acute{g}^{-1}(\Psi ^{-1}(\overset{\acute{t}}{\underset{\iota =1}{\sum }}\gamma _{\iota }\Psi (\acute{g}(\beta _{\iota }))))},\right. \\{} & {} \qquad \left. \left\{ \left( \Psi ^{-1}\left( \overset{\acute{t}}{\underset{\iota =1}{\sum }} \gamma _{\iota }\Psi (\Theta _{\iota }^{q})\right) \right) ^{{p_{i}}/q},\left( \Pi ^{-1}\left( \overset{\acute{t}}{\underset{\iota =1}{\sum }}\gamma _{\iota }\Pi (\varepsilon _{\iota })\right) \right) ^{p_{i}}, \left( \Pi ^{-1}\left( \overset{\acute{t}}{\underset{\iota =1}{\sum }}\gamma _{\iota }\Pi (\Pi _{\iota })\right) \right) ^{p_{i}}\right\} \right) \\{} & {} \qquad \oplus \left( \acute{s}_{\acute{g}^{-1}\left( \Psi ^{-1}\left( \gamma _{\acute{t}+1}\Psi (\acute{g}(\beta _{\acute{t}+1}))\right) \right) },\right. \\{} & {} \left. \qquad \left\{ \left( \Psi _{\acute{t}+1}^{-1}\left( \gamma _{\acute{t}+1} \Psi (\Theta _{\acute{t}+1}^{q})\right) \right) ^{^{p_{i}}/q},\left( \Pi ^{-1}\left( \gamma _{\acute{t}+1} \Pi (\varepsilon _{\acute{t}+1})\right) \right) ^{p_{i}},\left( \Pi ^{-1}\left( \gamma _{\acute{t}+1} \Pi (\Pi _{\acute{t}+1})\right) \right) ^{p_{i}}\right\} \right) \\{} & {} \quad = \left( \acute{s}_{\acute{g}^{-1}\left( \Psi ^{-1}\left( \overset{\acute{t}+1}{\underset{\iota =1}{\sum }}\gamma _{\iota }\Psi (\acute{g}(\beta _{\iota }))\right) \right) },\right. \\{} & {} \qquad \left. \left\{ \left( \Psi ^{-1}\left( \overset{\acute{t}+1}{\underset{\iota =1}{\sum }} \gamma _{\iota }\Psi (\Theta _{\iota }^{q})\right) \right) ^{{p_{i}}/q},\left( \Pi ^{-1}\left( \overset{\acute{t}+1}{\underset{\iota =1}{\sum }}\gamma _{\iota }\Pi (\varepsilon _{\iota })\right) \right) ^{p_{i}}, \left( \Pi ^{-1}\left( \overset{\acute{t}+1}{\underset{\iota =1}{\sum }}\gamma _{\iota }\Pi (\Pi _{\iota })\right) \right) ^{p_{i}}\right\} \right) , \end{aligned}$$
  The final result is fulfilled for $\Bbbk =\acute{t}+1.$

#### Theorem 4

(Idempotency) Let $\ddot{\delta }_{\iota }=\frac{p_{i}}{(\acute{s}_{\beta _{\iota }} ,\{\Theta _{\iota },\varepsilon _{\iota },\Pi _{\iota }\})}$, $(\iota =1,2,3,...,\Bbbk )$ and $(i=1,2,3,...,\Bbbk )$ are the array collection of P-QRLNS, here if all $\ddot{\delta }_{\iota }$ are the equals, i.e., $\ddot{\delta }_{\iota }=\ddot{\delta }\; \forall \; \iota$ and *p*, So:

$$\begin{aligned} P{\text{-}}QRLNWAA \left( \ddot{\delta }_{1},\ddot{\delta }_{2},...,\ddot{\delta }_{\Bbbk } \right) =\ddot{\delta }. \end{aligned}$$

#### *Proof*

From above, given that $\ddot{\delta }_{\iota }=\ddot{\delta }\; \forall \; \iota ,$ and *p* therefore:

$$\begin{aligned} P{\text{-}}QRLNWAA(\ddot{\delta }_{1},\ddot{\delta }_{2},\ddot{\delta }_{3},...,\ddot{\delta }_{\Bbbk })= & {} P{\text{-}}QRLNWAA(\ddot{\delta },\ddot{\delta },\ddot{\delta },...,\ddot{\delta })\\= & {} \left( \acute{s}_{\acute{g}^{-1}\left( \Psi ^{-1}\left( \overset{\Bbbk }{\underset{\iota =1}{\sum }}\gamma _{\iota }\Psi (\acute{g}(\beta _{\iota }))\right) \right) },\right. \\{} & {} \left. \left\{ \left( \Psi ^{-1}\left( \overset{\Bbbk }{\underset{\iota = 1}{\sum }} \gamma _{\iota }\Psi (\Theta _{\iota }^{q})\right) \right) ^{p_{i}/q},\left( \Pi ^{-1}\left( \overset{\Bbbk }{\underset{\iota = 1}{\sum }}\gamma _{\iota }\Pi (\varepsilon _{\iota })\right) \right) ^{p_{i}},\right. \right. \\{} & {} \left. \left. \left( \Pi ^{-1}\left( \overset{\Bbbk }{\underset{\iota = 1}{\sum }}\gamma _{\iota }\Pi (\Pi _{\iota })\right) \right) ^{p_{i}}\right\} \right) \\= & {} \left( \acute{s}_{\beta },\left\{ \Theta ,\varepsilon ,\Pi \right\} \right) = \ddot{\delta }. \end{aligned}$$

Hence, our result is proved. $\square$

#### Theorem 5

(Monotonicity) Let $\ddot{\delta }_{\iota }=\frac{p_{i}}{({s}_{\beta _{\iota }} ,\{\Theta _{\iota },\varepsilon _{\iota },\Pi _{\iota }\})}$ and $\Delta _{\iota }=\frac{p_{i}}{(\acute{s}_{\beta _{\iota }} ,\{\Theta _{\iota },\varepsilon _{\iota },\Pi _{\iota }\})}$, $(\iota =1,2,3,...,\Bbbk )$ and $(i=1,2,3,...,\Bbbk )$ are the collections of two P-QRLNS, If $\ddot{\delta }_{\iota }\le \Delta _{\iota }$, So:

$$\begin{aligned} P{\text{-}}QRLNWAA \left( \ddot{\delta }_{1},\ddot{\delta }_{2},...,\ddot{\delta }_{\iota }\right) \le P{\text{-}}QRLNWAA \left( \Delta _{1},\Delta _{2},...,\Delta _{l}\right) . \end{aligned}$$

#### *Proof*

Here is given, $g'$ is a monotonically increasing function. So,

$$\begin{aligned}{} & {} \left( \acute{s}_{\acute{g'}^{-1}(\Psi ^{-1}(\overset{\Bbbk }{\underset{\iota = 1}{\sum }}\gamma _{\iota }\Psi (\acute{g'}(\beta _{\iota }))))}, \left\{ \left( \Psi ^{-1}\left( \overset{\Bbbk }{\underset{\iota = 1}{\sum }} \gamma _{\iota }\Psi (\Theta _{\iota }^{q})\right) \right) ^{p_{i}/q}\right. \right. ,\\{} & {} \left. \left. \qquad \left( \Pi ^{-1}\left( \overset{\Bbbk }{\underset{\iota = 1}{\sum }}\gamma _{\iota }\Pi (\varepsilon _{\iota })\right) \right) ^{p_{i}}, \left( \Pi ^{-1}\left( \overset{\Bbbk }{\underset{\iota = 1}{\sum }}\gamma _{\iota }\Pi (\Pi _{\iota })\right) \right) ^{p_{i}}\right\} \right) \\{} & {} \quad \le \left( \acute{s}_{\acute{g'}^{-1}\left( \Psi ^{-1}\left( \overset{\Bbbk }{\underset{\iota = 1}{\sum }}\gamma _{\iota }\Psi (\acute{g'}(\beta _{\iota ^{*}}))\right) \right) }, \left\{ \left( \Psi ^{-1}\left( \overset{\Bbbk }{\underset{\iota =1}{\sum }} \gamma _{\iota }\Psi (\Theta _{\iota ^{*}}^{q})\right) \right) ^{p_{i}/q},\right. \right. \\{} & {} \left. \left. \qquad \left( \Pi ^{-1}\left( \overset{\Bbbk }{\underset{\iota =1}{\sum }}\gamma _{\iota }\Pi (\varepsilon _{\iota ^{*}})\right) \right) ^{p_{i}}, \left( \Pi ^{-1}\left( \overset{\Bbbk }{\underset{\iota = 1}{\sum }}\gamma _{\iota }\Pi (\Pi _{\iota ^{*}})\right) \right) ^{p_{i}}\right\} \right) \\{} & {} P{\text{-}}QRLNWAA \left( \ddot{\delta }_{1},\ddot{\delta }_{2},...,\ddot{\delta }_{\iota }\right) \le P{\text{-}}QRLNWAA \left( \Delta _{1},\Delta _{2},...,\Delta _{\iota }\right) . \end{aligned}$$

Hence, the prove is completed. $\square$

#### Theorem 6

(Boundedness) Suppose that $\ddot{\delta }_{\iota }=\frac{p_{i}}{({s}_{\beta _{\iota }} ,\{\Theta _{\iota },\varepsilon _{\iota },\Pi _{\iota }\})}$, $(\iota =1,2,3,...,\Bbbk )$ and $(i=1,2,3,...,\Bbbk )$ are array of P-QRLNS, So:

$$\begin{aligned} \ddot{\delta }_{\iota }^{-}\le P{\text{-}}QRLNWAA \left( \ddot{\delta }_{1},\ddot{\delta }_{2},...,\ddot{\delta }_{\Bbbk } \right) \le \ddot{\delta }_{\iota }^{+}, \end{aligned}$$

Here, $$\ddot{\delta }_{\iota }^{-}= \min \bigg (\ddot{\delta }_{1},\ddot{\delta }_{2},...,\ddot{\delta }_{\Bbbk }\bigg )$$ and $$\ddot{\delta }_{\iota }^{+}=\max \bigg (\ddot{\delta }_{1},\ddot{\delta }_{2},...,\ddot{\delta }_{\Bbbk }\bigg )$$.

#### *Proof*

Suppose that $c= min\bigg (\ddot{\delta }_{1},\ddot{\delta }_{2},...,\ddot{\delta }_{\Bbbk }\bigg )$ and $d= max\bigg (\ddot{\delta }_{1},\ddot{\delta }_{2},...,\ddot{\delta }_{\Bbbk }\bigg )$, using the Theorem 5, we have

$$\begin{aligned} \Rightarrow min\bigg (\ddot{\delta }_{1},\ddot{\delta }_{2},...,\ddot{\delta }_{\Bbbk }\bigg )\le & {} \bigg (\ddot{\delta }_{1},\ddot{\delta }_{2},...,\ddot{\delta }_{\Bbbk }\bigg )\le max\bigg (\ddot{\delta }_{1},\ddot{\delta }_{2},...,\ddot{\delta }_{\Bbbk }\bigg )\\ \Rightarrow c\le & {} \bigg (\ddot{\delta }_{1},\ddot{\delta }_{2},...,\ddot{\delta }_{\Bbbk }\bigg )\le d. \end{aligned}$$

Hence, it is the proof. $\square$

#### Theorem 7

(Symmetry) Let$\ddot{\delta }_{\iota }=\frac{p_{i}}{({s}_{\beta _{\iota }} ,\{\Theta _{\iota },\varepsilon _{\iota },\Pi _{\iota }\})}$, $(\iota =1,2,3,...,\Bbbk )$ and $(i=1,2,3,...,\Bbbk )$ are the P-QRLNS. If $\ddot{\delta }_{\iota ^{'}}=\frac{p_{i}}{(\acute{s}_{\beta _{\iota ^{'}}} ,\{\Theta _{\iota ^{'}},\varepsilon _{\iota ^{'}},\Pi _{\iota ^{'}}\})}$ be randomly permutation of $\ddot{\delta }_{\iota }=\frac{p_{i}}{(\acute{s}_{\beta _{\iota }} ,\{\Theta _{\iota },\varepsilon _{\iota },\Pi _{\iota }\})}$. Then, we have:

$$\begin{aligned} P{\text{-}}QRLNWAA\bigg (\frac{p_{i}}{(\acute{s}_{\beta _{\iota }},\{\Theta _{\iota },\varepsilon _{\iota },\Pi _{\iota }\})}\bigg ) =P{\text{-}}QRLNWAA \bigg (\frac{p_{i}}{(\acute{s}_{\beta _{\iota ^{'}}},\{\Theta _{\iota ^{'}},\varepsilon _{\iota ^{'}},\Pi _{\iota ^{'}}\})}\bigg ). \end{aligned}$$

#### *Proof*

This result is cleared. Therefore, it is omitted. $\square$

###  Probabilistic q-Rung linguistic neutrosophic number weighted geometric aggregation operator

#### Definition 17

^56^ Suppose that the $\ddot{\delta }_{\iota }=\frac{p_{i}}{(\acute{s}_{\beta _{\iota }} ,\{\Theta _{\iota },\varepsilon _{\iota },\Pi _{\iota }\})}$, $(\iota =1,2,3,...,\Bbbk )$ and $(i=1,2,3,...,\Bbbk )$ are arrays of P-QRLNS, the P-QRLNWGA operator is basing on the Archimedian t-conorm, t-norm and is follow as:

$$\begin{aligned} P{\text{-}}QRLNWGA(\ddot{\delta }_{1},\ddot{\delta }_{2},\ddot{\delta }_{3},...,\ddot{\delta }_{\Bbbk })= & {} \overset{\Bbbk }{\underset{\iota =1}{\otimes }} (\ddot{\delta }_{\iota })^{\gamma _{\iota }}. \end{aligned}$$

#### Theorem 8

Supposed that $\ddot{\delta }_{\iota }=\frac{p_{i}}{(\acute{s}_{\beta _{\iota }} ,\{\Theta _{\iota },\varepsilon _{\iota },\Pi _{\iota }\})}$, $(\iota =1,2,3,...,\Bbbk )$ and $(i=1,2,3,...,\Bbbk )$ based on the Archimedian norms with its types, we may define the following operator:

$$\begin{aligned} P{\text{-}}QRLNWGA(\ddot{\delta }_{1},\ddot{\delta }_{2},\ddot{\delta }_{3},...,\ddot{\delta }_{\Bbbk })= & {} \left( \acute{s}_{\acute{g}^{-1}(\Pi ^{-1}(\overset{\Bbbk }{\underset{\iota =1}{\sum }}\gamma _{\iota }\Pi (\acute{g}(\backepsilon _{\iota }))))},\right. \\{} & {} \left. \left\{ ,\left( \Pi ^{-1}\left( \overset{\Bbbk }{\underset{\iota =1}{\sum }}\gamma _{\iota }\Pi (\Theta _{\iota })\right) \right) , \left( \Pi ^{-1}\left( \overset{\Bbbk }{\underset{\iota =1}{\sum }}\gamma _{\iota }\Pi (\varepsilon _{\iota })\right) ,\right. \right. \right. \\{} & {} \left. \left. \left. \left( \Psi ^{-1}\left( \overset{\Bbbk }{\underset{\iota =1}{\sum }} \gamma _{\iota }\Psi (\Pi _{\iota }^{q})\right) \right) ^{1/q}\right) \right\} \right) . \end{aligned}$$

#### *Proof*

This theorem’s proof as similar to Theorem 3. So, we skipped because we can easily verify it. $\square$

Similar to the P-QRLNWGA operator, the P-QRLNWGA operator likewise possesses a number of intriguing characteristics, which are alleged (without evidence) as follows:

#### Theorem 9

(Idempotency) Let $\ddot{\delta }_{\iota }=\frac{p_{i}}{(\acute{s}_{\beta _{\iota }} ,\{\Theta _{\iota },\varepsilon _{\iota },\Pi _{\iota }\})}$, $(\iota =1,2,3,...,\Bbbk )$ and $(i=1,2,3,...,\Bbbk )$ be the arrays of P-QRLNS, If all $\ddot{\delta }_{\iota }$ are same, i.e., $\ddot{\delta }_{\iota }=\ddot{\delta }\; \forall \; \iota ,$ and *p* So:

$$\begin{aligned} P{\text{-}}QRLNWGA \left( \ddot{\delta }_{1},\ddot{\delta }_{2},...,\ddot{\delta }_{\Bbbk } \right) =\ddot{\delta }. \end{aligned}$$

#### Theorem 10

(Monotonicity) Let $\ddot{\delta }_{\iota }=\frac{p_{i}}{(\acute{s}_{\beta _{\iota }} ,\{\Theta _{\iota },\varepsilon _{\iota },\Pi _{\iota }\})}$, and $\Delta _{\iota }=\frac{p_{i}}{(\acute{s}_{\beta _{\iota }} ,\{\Theta _{\iota },\varepsilon _{\iota },\Pi _{\iota }\})}$ are the P-QRLNS, If $\ddot{\delta }_{\iota }\le \Delta _{\iota }$,

$$\begin{aligned} P{\text{-}}QRLNWGA \left( \ddot{\delta }_{1},\ddot{\delta }_{2},...,\ddot{\delta }_{\iota }\right) \le P{\text{-}}QRLNWAA \left( \Delta _{1},\Delta _{2},...,\Delta _{\iota }\right) . \end{aligned}$$

#### Theorem 11

(Boundedness) Let $\ddot{\delta }_{\iota }=\frac{p_{i}}{(\acute{s}_{\beta _{\iota }} ,\{\Theta _{\iota },\varepsilon _{\iota },\Pi _{\iota }\})}$, $(\iota =1,2,3,...,\Bbbk )$ and $(i=1,2,3,...,\Bbbk )$ are P-QRLNS, So,

$$\begin{aligned} \ddot{\delta }_{\iota }^{-}\le P{\text{-}}RLNWGA \left( \ddot{\delta }_{1},\ddot{\delta }_{2},...,\ddot{\delta }_{\Bbbk } \right) \le \ddot{\delta }_{\iota }^{+}. \end{aligned}$$

#### Theorem 12

(Symmetry) Suppose that$\ddot{\delta }_{\iota }=\frac{p_{i}}{(\acute{s}_{\beta _{\iota }} ,\{\Theta _{\iota },\varepsilon _{\iota },\Pi _{\iota }\})}$, $(\iota =1,2,3,...,\Bbbk )$ and $(i=1,2,3,...,\Bbbk )$ be the P-QRLNS collections. If $\ddot{\delta }_{\iota ^{'}}=\frac{p_{i}}{(\acute{s}_{\beta _{\iota ^{'}}} ,\{\Theta _{\iota ^{'}},\varepsilon _{\iota ^{'}},\Pi _{\iota ^{'}}\})}$ be randomly permutation of $\ddot{\delta }_{\iota }=\frac{p_{i}}{(\acute{s}_{\beta _{\iota }} ,\{\Theta _{\iota },\varepsilon _{\iota },\Pi _{\iota }\})}$, Then:

$$\begin{aligned} P{\text{-}}QRLNWGA\bigg (\frac{p_{i}}{(\acute{s}_{\beta _{\iota }},\{\Theta _{\iota },\varepsilon _{\iota },\Pi _{\iota }\})}\bigg ) =P{\text{-}}QRLNWGA \bigg (\frac{p_{i}}{(\acute{s}_{\beta _{\iota ^{'}}},\{\Theta _{\iota ^{'}},\varepsilon _{\iota ^{'}},\Pi _{\iota ^{'}}\})}\bigg ). \end{aligned}$$

$$\square$$

## An innovative technique for effective decision-making

**Figure 1:**
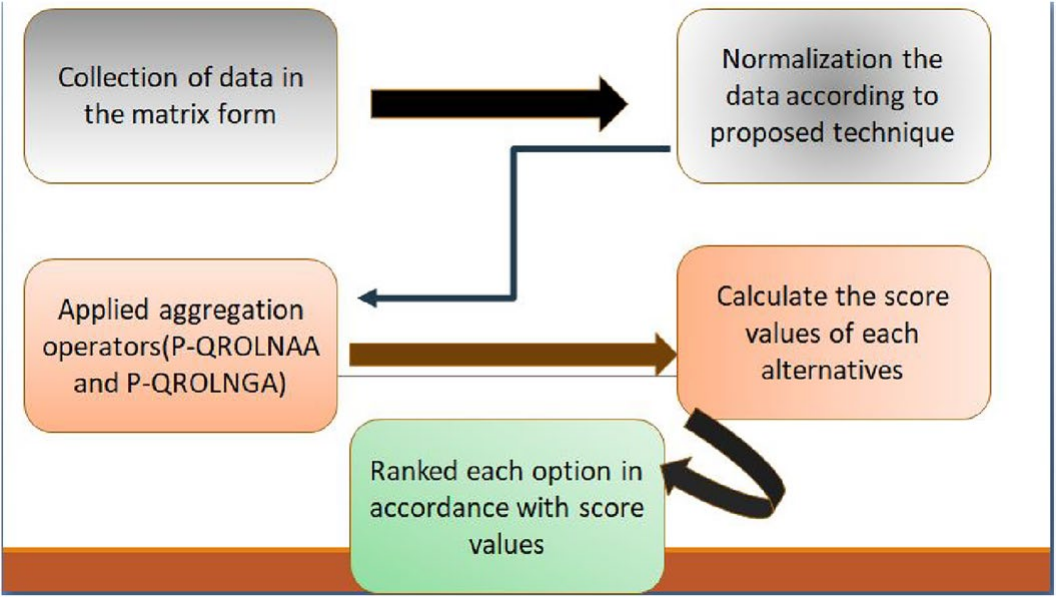
Flow diagram for the proposed method.

In this part, we have outlined a method for solving MADM issues that is based on P-QRLNS operators according to the algorithm in Fig. 1. Let’s say we have N = $\{n_{1}, n_{2}, n_{3},..., n_{k}\}$ be any finite arrays of *k* alternative and we have finite set of attribute such as S = $\{s_{1},s_{2},s_{3},...,s_{\iota }\}$. Dealing with qualitative entities (variables), such as enormous, extremely large, immense, etc., in DM is sometimes challenging. As a result, these entities must take into account numerical quantities. A linguistic variable and probabilistic degree, which functions as a type of mapping between a collection of linguistic things to a certain range of real numbers, is used to address such variables. For instance, Chatterjee et al.^57^ regarded the “quality of product” to be a linguistic variable. Using the probabilistic q-rung linguistic neutrosophic set as a foundation, they have gathered the data in the form of $\Delta =\frac{p}{(s_{\backepsilon }, \{\Theta ,\Psi ,\Pi \})}$ where, $s_{\backepsilon }$ is taken from linguistic set $S=\{s_{0}$ = Extremely bad, $s_{1}$ = Dreadful, $s_{2}$ = Poor, $s_{3}$ = Unbiased/Fair, $s_{4}$ = Excellent/Outstanding, $s_{5}$ = All right} and the condition for quantitative part of $\Delta$ is $0\le \Theta ^{q}+\Psi ^{q}+\Pi ^{q}\le 3$ and $p \in [0, 1]$.

1. Data collection:
  Collect evaluation data from the decision-makers in the type of a matrix G = $[{{\text{N}}}_{nm}]$ as,
  $$\begin{aligned} \text{G}=\left( \begin{array}{cccc} \text{N}_{11} &{} \text{N}_{12} &{} \cdots &{} \text{N}_{1m} \\ \text{N}_{21} &{} \text{N}_{22} &{} \cdots &{} \text{N}_{2m} \\ \vdots &{} \vdots &{} \cdots &{} \vdots \\ \text{N}_{n1} &{} \text{N}_{n2} &{} \cdots &{} \text{N}_{nm} \\ \end{array} \right) \end{aligned}$$
2. Normalization:
  The decision matrix is used in this stage as ${\mathcal {G}}=[{\mathcal {N}}_{xy}]$ into the normalized matrix transformation $\bar{{\mathcal {G}}}=[\bar{{\mathcal {N}}_{xy}}]$ by the given calculation method:
  $$\begin{aligned} \mathcal {{\bar{N}}}_{xy} ={\left\{ \begin{array}{ll} {\mathcal {N}}_{xy}, &{} \text{ if } \text{ it } \text{ is } \text{ from } \text{ Benefit-attribute } , \\ ({\mathcal {N}}_{xy})^{c}, &{} \text{ if } \text{ it } \text{ is } \text{ from } \text{ Cost-attribute } . \end{array}\right. } \end{aligned}$$
  here ${{\mathcal {N}}_{xy}^{c}}$ is referred as complement of ${\mathcal {N}}_{xy}$. Worth noting is the fact that for every q-RLN ${\mathcal {N}} =\frac{p}{(s_{\backepsilon }, \{\Pi ,\Psi ,\Theta \})}$ its complement can be calculated as,
  3 $$\begin{aligned} {\mathcal {B}}^{c}=\frac{p}{(s_{\backepsilon }, \{\Pi ,\Psi ,\Theta \})}. \end{aligned}$$
3. Aggregation:
  Aggregate the P-q-RLNs $\text{N}_{xy}$(y = 1, 2, 3,..., p) for all alternative $N_{x}(x=1, 2, 3,..., q)$ into the overall worth of preference $\text{N}$ by using the P-q-RLNAA or P-q-RLNGA operators that have been suggested.
  In mathematics, it may be expressed as;
  $$\begin{aligned} \text{N}_{x}= & {} P{\text{-}}q{\text{-}}RLNsAA_{\delta '}(\text{N}_{x1},\text{N}_{x2},\text{N}_{x3},...,\text{N}_{xp}),\\ \text{N}_{x}= & {} P{\text{-}}q{\text{-}}RLNsGA_{\delta '}(\text{N}_{x1},\text{N}_{x2},\text{N}_{x3},...,\text{N}_{xp}), \end{aligned}$$
  where $\gamma '=(\gamma '_{1},\gamma '_{2},...,\gamma '_{n})$ is the attributes of probabilistic-vector.
4. Identification of the score values:
  According to Eq.(2), find out the score values Sc($\text{N}_{x})$(x = 1, 2, 3, ..., p) of all P-q-RLNs $\text{N}_{x}$(x = 1, 2, 3, ..., p).
5. Main results with ranking:
  Sort the options into order to find the best one. $t_{x}(x=1, 2, 3, .., p)$ using the score values Scr($\text{N}_{x})$.

## Explanatory example

In this section, an explanatory example regarding the process of ranking of different nanoparticles is used to elaborate on the implications and practicality of the suggested approach. The ranking of nanoparticles is an important measure of their performance in the industries and other fields. These engineered nanoprticles offer promising benefits in various industries such as automotive Industry, electronics industry, aerospace industry, medical industry, oil and gas industry, HVAC (Heating, Ventilation, and Air Conditioning) and environmental remediation. These are some examples of industries and applications where nanoparticles are currently used or have the potential for use. The specific applications and industries may continue to evolve as nanotechnology research advances and new opportunities emerge. By incorporating nanoparticles exhibit improved thermal conductivity, leading to enhanced heat transfer capabilities. This property enables efficient cooling and heating in applications such as electronics, automotive systems, and renewable energy technologies. Additionally, nanoparticles can be tailored to specific applications, ensuring versatility across industries. However, the production cost, material compatibility, and safety considerations associated with nanoparticles pose challenges. Balancing the benefits and costs of nanoparticles is essential for their successful implementation and commercial viability. These parameters include some benefits parameters such as thermal conductivity enhancement, heat transfer performance, stability, application versatility, material compatibility and some cost parameters such as nanoparticle cost, manufacturing cost, safety considerations, scale-up and production efficiency. It is noted that the majority of nanoparticles are examined using the associated standards: Thermal conductivity enhancement $({\mathcal {S}}_1)$, Heat transfer performance$({\mathcal {S}}_2)$, Material Compatability $({\mathcal {S}}_3)$, Stability $({\mathcal {S}}_4)$, Nanoparticle cost $({\mathcal {S}}_5)$ and Manufacturing cost $({\mathcal {S}}_6)$.

Here are a few examples of nanoparticles that have shown promise in various applications:

- *Copper oxide* Copper oxide nanoparticles have been widely studied for their enhanced thermal conductivity properties. They can be used in heat transfer fluids for applications like electronics cooling and solar thermal systems.
- *Alumina* Alumina (aluminum oxide) nanoparticles are known for their stability and relatively low cost. They find applications in both heat transfer and lubrication enhancement.
- *Graphene* Graphene, a two-dimensional carbon allotrope, has exceptional thermal and electrical conductivity. Graphene nanoparticles have potential applications in electronics cooling and thermal management.
- *Carbon nanotube* Carbon nanotubes possess excellent mechanical, thermal, and electrical properties. They can be used in heat transfer fluids and as additives for enhancing thermal properties.
- *Magnetic* These magnetic nanoparticles are used in applications such as magnetic cooling and targeted drug delivery.
- *Titanium dioxide* Titanium dioxide nanoparticles have photocatalytic properties and find use in applications like solar collectors and wastewater treatment.
- *Silver* Silver nanoparticles have antimicrobial properties and are used in medical applications, such as wound healing and antibacterial coatings.
- *Silica* Silica nanoparticles are chemically stable and can be used in various applications, including electronics cooling and solar collectors.

The best nanoparticles varies depending on factors like the intended application, desired properties (e.g., thermal conductivity enhancement, stability), cost considerations, and safety concerns. It’s crucial to evaluate nanoparticles based on their performance in your specific context rather than seeking a one-size-fits-all best nanoparticle. Researchers and engineers often conduct experiments and tests to determine the most suitable nanoparticle for their intended purpose.

Then, at that point how industries choose the best nanoparticles in which five top rated ranking of nanoparticles are following such as: Metal oxide $({\mathcal {N}}_1)$, Carbon nanotube (CNT) $({\mathcal {N}}_2 )$, Magnetic nanoparticle $({\mathcal {N}}_3)$, Graphene $({\mathcal {N}}_4)$ and Metallic $({\mathcal {N}}_5)$. Clearly the determination interaction of nanoparticles is a MCDM issue comprise of five options $\left\{ n_1, n_2, n_3, n_4, n_5\right\}$, six models $\left\{ s_1, s_2, s_3, s_4, s_5, s_6 \right\}$ and specialist *d*.

For linguistic term, we have used the value of *t* by taking $t=6$. Then the linguistics terms and neutrosophic number for each alternatives and parameters in the form of set is as follow:

The suggested framework for choosing nanoparticles is novel, although it is not beyond limitations. Large datasets might be difficult for the model to handle, which could affect computing performance. Furthermore, the use of linguistic quantification might lead to subjectivity, and the complexity of the aggregation operator could make implementation in practice difficult. To solve these issues and improve the model’s use in various industrial contexts, ongoing validation and improvement are essential.

Step 1: Data collection in the matrix form (For q = 2) by using Eq. (1) as shown in Table 1.
  We obtained the data by employing specific ranking criteria. The process involved collecting rough data, and during this stage, we adhered to our predefined criteria to ensure the data aligns with our proposed definition of a probabilistic q-rung orthopair linguistic neutrosophic set. The reliability of the data, indicating its consistency and dependability, was maintained through rigorous adherence to these criteria. Additionally, the validity of the data, ensuring it accurately reflects our intended measurements, was upheld by consistently applying the specified criteria throughout the data collection process.
  The process of creating the tables, which involved ranking alternatives row-wise based on specific criteria. Additionally, we outlined the attributes used for ranking alternatives column-wise.

**Table 1:** Decision-matrix of probabilistic q-rung orthopair linguistic neutrosophic set taken by *D*.

	${\mathcal {S}}_1$	${\mathcal {S}}_2$	${\mathcal {S}}_3$	${\mathcal {S}}_4$	${\mathcal {S}}_5$	${\mathcal {S}}_6$
${\mathcal {N}}_{1}$	$\frac{0.8}{\langle s_{5},\{0.8,0.6,0.4\}\rangle }$	$\frac{0.9}{\langle s_{3},\{0.8,0.5,0.5\}\rangle }$	$\frac{0.5}{\langle s_{4},\{0.7,0.5,0.5\}\rangle }$	$\frac{0.6}{\langle s_{4},\{0.9,0.4,0.5\}\rangle }$	$\frac{0.4}{\langle s_{3},\{0.4,0.6,0.9\}\rangle }$	$\frac{0.7}{\langle s_{2},\{0.2,0.4,0.8\}\rangle }$
${\mathcal {N}}_2$	$\frac{0.6}{\langle s_{4},\{0.7,0.4,0.4\}\rangle }$	$\frac{0.7}{\langle s_{5},\{0.9,0.2,0.3\}\rangle }$	$\frac{0.8}{\langle s_{4},\{0.9,0.4,0.4\}\rangle }$	$\frac{0.6}{\langle s_{4},\{0.8,0.5,0.4\}\rangle }$	$\frac{0.7}{\langle s_{2},\{0.4,0.6,0.9\}\rangle }$	$\frac{0.8}{\langle s_{3},\{0.3,0.6,0.9\}\rangle }$
${\mathcal {N}}_3$	$\frac{0.7}{\langle s_{4},\{0.7,0.6,0.4\}\rangle }$	$\frac{0.7}{\langle s_{3},\{0.7,0.4,0.6\}\rangle }$	$\frac{0.4}{\langle s_{4},\{0.8,0.4,0.5\}\rangle }$	$\frac{0.6}{\langle s_{4},\{0.8,0.3,0.4\}\rangle }$	$\frac{0.4}{\langle s_{1},\{0.4,0.6,0.9\}\rangle }$	$\frac{0.4}{\langle s_{2},\{0.4,0.6,0.9\}\rangle }$
${\mathcal {N}}_4$	$\frac{0.8}{\langle s_{3},\{0.7,0.4,0.4\}\rangle }$	$\frac{0.6}{\langle s_{5},\{0.8,0.4,0.5\}\rangle }$	$\frac{0.7}{\langle s_{4},\{0.7,0.4,0.5\}\rangle }$	$\frac{0.6}{\langle s_{3},\{0.6,0.4,0.4\}\rangle }$	$\frac{0.6}{\langle s_{1},\{0.5,0.4,0.9\}\rangle }$	$\frac{0.5}{\langle s_{2},\{0.2,0.3,0.9\}\rangle }$
${\mathcal {N}}_5$	$\frac{0.8}{\langle s_{2},\{0.8,0.4,0.4\}\rangle }$	$\frac{0.5}{\langle s_{4},\{0.9,0.4,0.5\}\rangle }$	$\frac{0.9}{\langle s_{3},\{0.6,0.3,0.2\}\rangle }$	$\frac{0.6}{\langle s_{3},\{0.7,0.4,0.4\}\rangle }$	$\frac{0.6}{\langle s_{2},\{0.4,0.5,1.0\}\rangle }$	$\frac{0.8}{\langle s_{1},\{0.4,0.6,0.9\}\rangle }$
Step 2: Normalize the above given data by using Eq.(3) as shown in Table 2.

**Table 2:** Normalized matrix.

	${\mathcal {S}}_1$	${\mathcal {S}}_2$	${\mathcal {S}}_3$	${\mathcal {S}}_4$	${\mathcal {S}}_5$	${\mathcal {S}}_6$
${\mathcal {N}}_{1}$	$\frac{0.8}{\langle s_{5},\{0.8,0.6,0.4\}\rangle }$	$\frac{0.9}{\langle s_{3},\{0.8,0.5,0.5\}\rangle }$	$\frac{0.5}{\langle s_{4},\{0.7,0.5,0.5\}\rangle }$	$\frac{0.6}{\langle s_{4},\{0.9,0.4,0.5\}\rangle }$	$\frac{0.4}{\langle s_{3},\{0.9,0.6,0.4\}\rangle }$	$\frac{0.7}{\langle s_{2},\{0.8,0.4,0.2\}\rangle }$
${\mathcal {N}}_2$	$\frac{0.6}{\langle s_{4},\{0.7,0.4,0.4\}\rangle }$	$\frac{0.7}{\langle s_{5},\{0.9,0.2,0.3\}\rangle }$	$\frac{0.8}{\langle s_{4},\{0.9,0.4,0.4\}\rangle }$	$\frac{0.6}{\langle s_{4},\{0.8,0.5,0.4\}\rangle }$	$\frac{0.7}{\langle s_{2},\{0.9,0.6,0.4\}\rangle }$	$\frac{0.8}{\langle s_{3},\{0.9,0.6,0.3\}\rangle }$
${\mathcal {N}}_3$	$\frac{0.7}{\langle s_{4},\{0.7,0.6,0.4\}\rangle }$	$\frac{0.7}{\langle s_{3},\{0.7,0.4,0.6\}\rangle }$	$\frac{0.4}{\langle s_{4},\{0.8,0.4,0.5\}\rangle }$	$\frac{0.6}{\langle s_{4},\{0.8,0.3,0.4\}\rangle }$	$\frac{0.4}{\langle s_{1},\{0.9,0.6,0.4\}\rangle }$	$\frac{0.4}{\langle s_{2},\{0.9,0.6,0.4\}\rangle }$
${\mathcal {N}}_4$	$\frac{0.8}{\langle s_{3},\{0.7,0.4,0.4\}\rangle }$	$\frac{0.6}{\langle s_{5},\{0.8,0.4,0.5\}\rangle }$	$\frac{0.7}{\langle s_{4},\{0.7,0.4,0.5\}\rangle }$	$\frac{0.6}{\langle s_{3},\{0.6,0.4,0.4\}\rangle }$	$\frac{0.6}{\langle s_{1},\{0.9,0.4,0.4\}\rangle }$	$\frac{0.5}{\langle s_{2},\{0.9,0.3,0.2\}\rangle }$
${\mathcal {N}}_5$	$\frac{0.8}{\langle s_{2},\{0.8,0.4,0.4\}\rangle }$	$\frac{0.5}{\langle s_{4},\{0.9,0.4,0.5\}\rangle }$	$\frac{0.9}{\langle s_{3},\{0.6,0.3,0.2\}\rangle }$	$\frac{0.6}{\langle s_{3},\{0.7,0.4,0.4\}\rangle }$	$\frac{0.6}{\langle s_{2},\{1.0,0.5,0.4\}\rangle }$	$\frac{0.8}{\langle s_{1},\{0.9,0.6,0.4\}\rangle }$
Step 3: We used aggregation operators in this step (P-q-RLNWAA and P-q-RLNWGA) by using known weights $\{0.2413,0.1972,0.1793,0.1973,0.1849\}$ which we have from the prior step.
  We obtained results:
  - According to Theorem 3, P-q-RLNWAA :
    $${\mathcal {N}}_{1}=(s_{3.5953},\{0.3847,0.4913,0.5770\})$$, $${\mathcal {N}}_{2}=(s_{3.0614},\{0.3748,0.5304,0.3715\})$$,
    $${\mathcal {N}}_{3}=(s_{2.1610},\{0.4101,0.5398,0.4682\})$$, $${\mathcal {N}}_{4}=(s_{2.6898},\{0.5120,0.5805,0.4535\})$$, and
    $${\mathcal {N}}_{5}=(s_{2.4705},\{0.5416,0.6523,0.5623\})$$.
  - According to Theorem 8, P-q-RLNWGA :
    $${\mathcal {N}}_{1}=(s_{3.3247},\{0.2911,0.4993,0.7297\})$$, $${\mathcal {N}}_{2}=(s_{2.3917},\{0.3251,0.5304,0.7575\})$$,
    $${\mathcal {N}}_{3}=(s_{1.8233},\{0.3427,0.5398,0.6835\})$$, $${\mathcal {N}}_{4}=(s_{2.2244},\{0.4548,0.5805,0.7196\})$$, and
    $${\mathcal {N}}_{5}=(s_{1.8464},\{0.4313,0.6523,0.6712\})$$.
Step 4: In this step, we calculated the values of score function for each alternatives by using Eq.(2).
  - P-q-RLNWAA:
    $$Sc({\mathcal {N}}_{1})=2.9304$$, $$Sc({\mathcal {N}}_{2})=3.0689$$, $$Sc({\mathcal {N}}_{3})=2.0507$$, $$Sc({\mathcal {N}}_{4})=2.8417$$ and $$Sc({\mathcal {N}}_{5})=2.4107$$.
  - P-q-RLNWGA:
    $$Sc({\mathcal {N}}_{1})=1.8361$$, $$Sc({\mathcal {N}}_{2})=1.7821$$, $$Sc({\mathcal {N}}_{3})=1.1856$$, $$Sc({\mathcal {N}}_{4})=1.5326$$ and $$Sc({\mathcal {N}}_{5})=1.3580$$.

**Figure 2:**
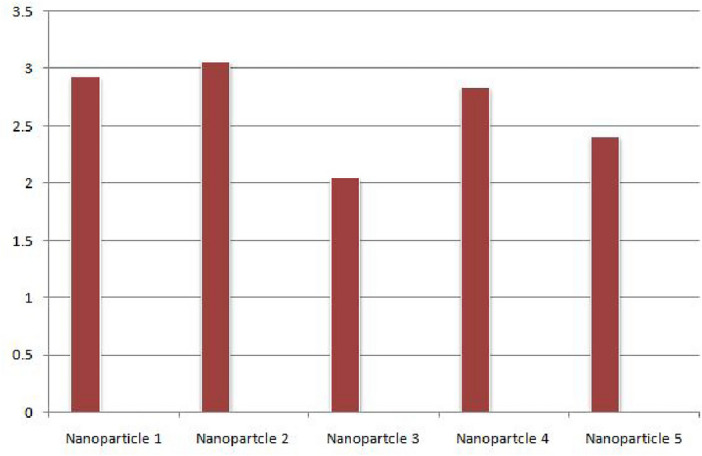
Using P-q-RLNWAA, graphically ranking of nanoparticles.

**Figure 3:**
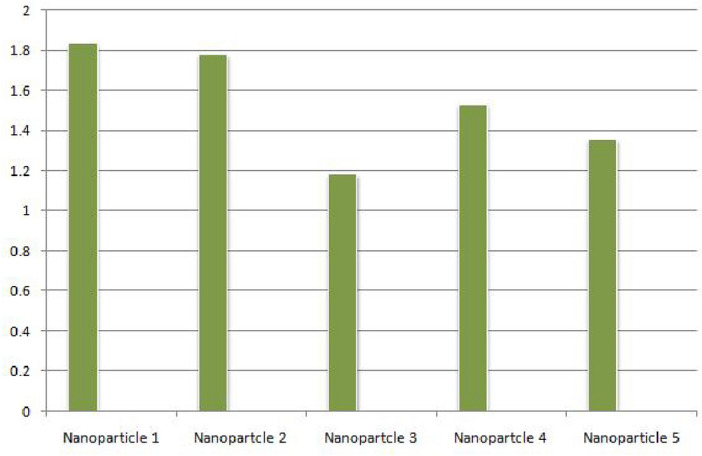
Using P-q-RLNWGA, graphically ranking of nanoparticles.
Step 5: Then, we assigned a score to each alternative.
  - P-q-RLNWAA:
    $$\begin{aligned} {\mathcal {N}}_{2}>{\mathcal {N}}_{1}>{\mathcal {N}}_{4}>{\mathcal {N}}_{5}>{\mathcal {N}}_{3}. \end{aligned}$$
  - P-q-RLNWGA:
    $$\begin{aligned} {\mathcal {N}}_{1}>{\mathcal {N}}_{2}>{\mathcal {N}}_{4}>{\mathcal {N}}_{5}>{\mathcal {N}}_{3}. \end{aligned}$$
    The aggregation operators display the finished ranks as a result. Magnetic nanoparticles have the highest rating among nanoparticles according to the P-q-RLNWAA, which demonstrates the supremacy of nanoparticles as shown in Fig. 2. However, P-q-RLNWGA demonstrates that when compared to other nanoparticles, nanotubes have the greatest rating as shown in Fig. 3. The outcomes for both operators are too similar but produce results that are average.
    The list’s order suggests a hierarchy of nanoparticle quality in the realm of fluid dynamics and heat transfer. However, the inferences made speculative without particular knowledge on the fluids these labels reflect. It is advised to check reputable sources from industry for accurate and current information because nanoparticle ranks in industrial areas might fluctuate. The industry rankings offer thorough and trustworthy evaluations of the performances and ranks of nanoparticles, giving accurate information for fluids aficionados.

## Managerial and policy implications for proposed technique

Some points of managerial and policy implications for our proposed method are as following:

1. *Resource optimization and cost efficiency* Implementing the proposed framework enables industries to optimize resources effectively. Managers can strategically select nanoparticles based on the probabilistic q-rung orthopair linguistic neutrosophic quantification, leading to cost-efficient operations. This approach assists in minimizing wastage and ensuring that resources are allocated judiciously.
2. *Enhanced decision-making process* The framework provides a systematic and structured approach to decision-making in nanoparticle selection. Managers can use the probabilistic q-rung orthopair linguistic neutrosophic quantification to evaluate options more comprehensively, leading to informed decisions. This ensures that the chosen nanoparticles align with the specific needs and goals of the industry.
3. *Risk management and contingency planning* Inherent uncertainties in the industrial environment necessitate a robust risk management strategy. The probabilistic aspect of the framework allows for the incorporation of uncertainties into decision-making. Managers can assess potential risks associated with different nanoparticles and develop contingency plans to mitigate adverse effects on production and operations.
4. *Compliance and regulatory alignment* Industries are subject to various regulations and standards. The proposed framework helps managers ensure that the selected nanoparticles align with regulatory requirements. This enhances compliance and reduces the risk of legal issues. Additionally, a structured approach to decision-making facilitates transparent communication with regulatory bodies.
5. *Sustainable practices and environmental impact* The framework promotes the consideration of sustainability criteria in nanoparticle selection. Managers can assess the environmental impact of different nanoparticles and choose options that align with sustainability goals. This approach contributes to the overall corporate social responsibility of the industry and may lead to positive public perception.
6. *Continuous improvement and adaptability* The dynamic nature of industries requires continuous improvement and adaptability. Managers can use the framework to regularly reassess and adapt nanoparticle selection strategies based on changing industry trends, technological advancements, and market dynamics. This ensures that the industry remains competitive and resilient over time.
7. *Stakeholder engagement and communication* Clear and effective communication is vital for successful implementation. Managers can use the framework to communicate decisions regarding nanoparticle selection to various stakeholders, including employees, investors, and customers. This fosters transparency and helps build trust among stakeholders.
8. *Training and skill development* Implementing the proposed framework may require training employees on the concepts of probabilistic q-rung orthopair linguistic neutrosophic quantification. Investing in training programs enhances the skillset of the workforce, ensuring that the framework is applied effectively and consistently across the organization.
9. *Long-term strategic planning* The framework supports long-term strategic planning by providing a structured methodology for nanoparticle selection. Managers can align nanoparticle decisions with the overall strategic goals of the industry, contributing to sustained growth and competitiveness.
10. *Benchmarking and performance measurement* Establishing benchmarks and key performance indicators (KPIs) related to nanoparticle selection allows managers to assess the effectiveness of the framework over time. Regular performance measurement ensures that the industry continues to achieve its objectives and can identify areas for improvement.

## Sensitivity analysis

A type of monetary model called sensitivity analysis impacts of changes in input factors on track factors. It is a technique for anticipating a choice’s result given a bunch of significant factors. Sensitivity analysis is used to handle the uncertainty in mathematical models, when the values for the model’s inputs may fluctuate. The two are usually used in combination since it is the analytical technique that goes along with uncertainty analysis. All models constructed and studies done rely on assumptions regarding the accuracy of the inputs used in calculations to obtain results or conclusions for policy decisions. Sensitivity analysis might be useful in various circumstances, including estimating, anticipating, and recognizing regions that need cycle upgrades or changes. However, utilizing historical data might occasionally result in incorrect projections since past occurrences don’t necessarily foretell future ones.

### “q” parameter sensitivity

#### “P-QRLNWAA” operator

**Figure 4:**
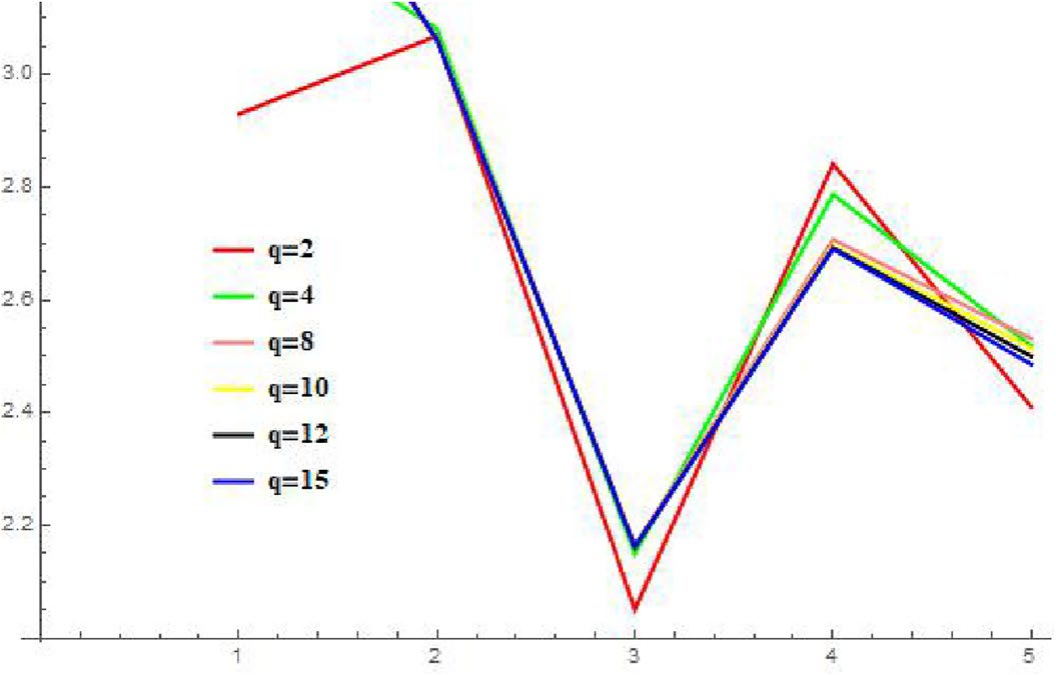
Using P-q-RLNWAA, Graphically representation of sensitivity analysis with regard to the parameter q.

In this section, to explore the impact of different q parameter values on the ranking of the other choices, we simply do the responsiveness analysis using the P-QRLNWAA operator in Table 3, and the chart shows that when we increase the values of q, very nothing really changes. Additionally, we have observed that when the values of q grew, the values of score function of each choice became more modest. The optimal choice remains the same when q = 2, q = 4, and q = 8, but it changes when q = 10, q = 12, and q = 15 are entered. Additionally, we have observed that how each option behaved by looking at the graphic representation of its score-values in Fig. 4. There is a very small change is appearing in Fig. 2. The parameter q is like a representation of the DM’s attitude. The aggregation operator is appropriate when dealing with critical decision-makers, while the P-QRLNWAA operator is helpful when reflecting optimistic decision-makers. Suppose we use the P-QRLNWAA operator to collect data for the current cycle. In that case, higher q values indicate that decision-makers have a more negative attitude, while lower values indicate a more positive attitude. Therefore, different DM can select the most appropriate value of q based on their attitude.

**Table 3:** A different ranking by altering the parameter values.

q-values	Values of score function	Ranking
q = 2	$Scr({\mathcal {N}}_{1})=2.9307$, $Scr({\mathcal {N}}_{2})=3.0678$, $Scr({\mathcal {N}}_{3})=2.0509$, $Scr({\mathcal {N}}_{4})=2.8414$, $Scr({\mathcal {N}}_{5})=2.4106$	${\mathcal {N}}_{2}>{\mathcal {N}}_{1}>{\mathcal {N}}_{4}>{\mathcal {N}}_{5}>{\mathcal {N}}_{3}$
q = 4	$Scr({\mathcal {N}}_{1})=3.3067$, $Scr({\mathcal {N}}_{2})=3.0815$, $Scr({\mathcal {N}}_{3})=2.1495$, $Scr({\mathcal {N}}_{4})=2.7866$, $Scr({\mathcal {N}}_{5})=2.5188$	${\mathcal {N}}_{1}>{\mathcal {N}}_{2}>{\mathcal {N}}_{4}>{\mathcal {N}}_{5}>{\mathcal {N}}_{3}$
q = 8	$Scr({\mathcal {N}}_{1})=3.5564$, $Scr({\mathcal {N}}_{2})=3.0635$, $Scr({\mathcal {N}}_{3})=2.1656$, $Scr({\mathcal {N}}_{4})=2.7065$, $Scr({\mathcal {N}}_{5})=2.5356$	${\mathcal {N}}_{1}>{\mathcal {N}}_{2}>{\mathcal {N}}_{4}>{\mathcal {N}}_{5}>{\mathcal {N}}_{3}$
q = 10	$Scr({\mathcal {N}}_{1})=3.5825$, $Scr({\mathcal {N}}_{2})=3.0626$, $Scr({\mathcal {N}}_{3})=2.1636$, $Scr({\mathcal {N}}_{4})=2.6967$, $Scr({\mathcal {N}}_{5})=2.5146$	${\mathcal {N}}_{1}>{\mathcal {N}}_{2}>{\mathcal {N}}_{4}>{\mathcal {N}}_{5}>{\mathcal {N}}_{3}$
q = 12	$Scr({\mathcal {N}}_{1})=3.5908$, $Scr({\mathcal {N}}_{2})=3.0616$, $Scr({\mathcal {N}}_{3})=2.1617$, $Scr({\mathcal {N}}_{4})=2.6926$, $Scr({\mathcal {N}}_{5})=2.5055$	${\mathcal {N}}_{1}>{\mathcal {N}}_{2}>{\mathcal {N}}_{4}>{\mathcal {N}}_{5}>{\mathcal {N}}_{3}$
q = 15	$Scr({\mathcal {N}}_{1})=3.5946$, $Scr({\mathcal {N}}_{2})=3.0619$, $Scr({\mathcal {N}}_{3})=2.1617$, $Scr({\mathcal {N}}_{4})=2.6909$, $Scr({\mathcal {N}}_{5})=2.4868$	${\mathcal {N}}_{1}>{\mathcal {N}}_{2}>{\mathcal {N}}_{4}>{\mathcal {N}}_{5}>{\mathcal {N}}_{3}$

#### “P-QRLNWGA” operator

**Figure 5:**
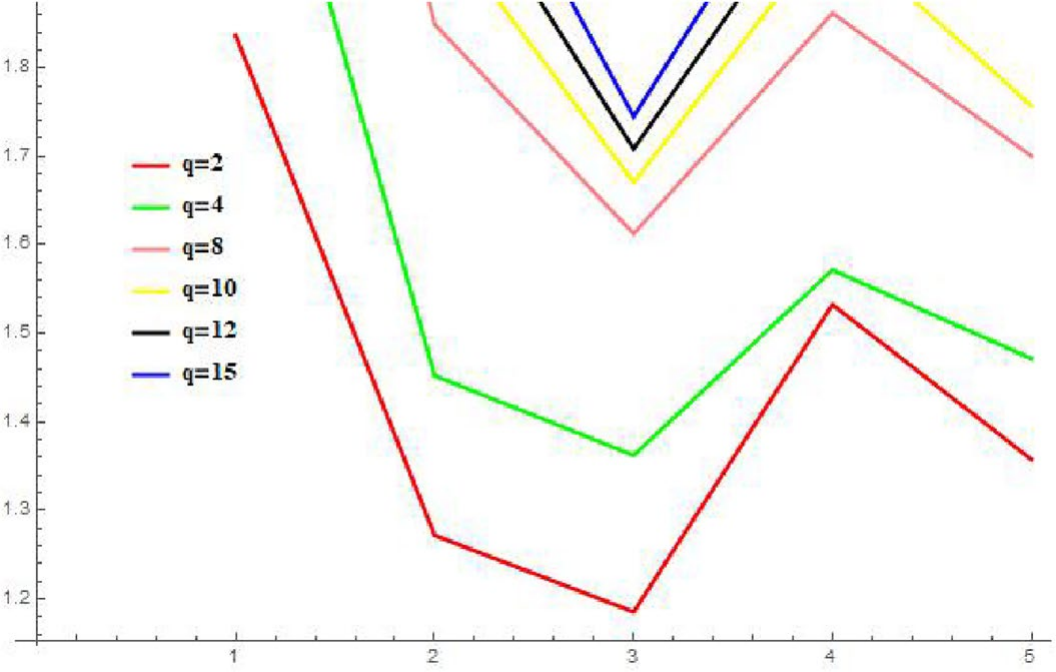
Using P-q-RLNGAA, Graphically representation of sensitivity analysis with regard to the parameter q.

In this section, we used P-QRLNWGA to adjust the values of the parameter “q” and saw how the ranking of the alternative changed. There is almost no probabilistic that occurs, similarly to the P-QRLNWAA operator, as the value of the parameter change should be obvious in Table 4. Adding to the conduct of score values, assuming equality, can be seen in Fig. 5, where minimal change is occurring when q’s values alter. In order for DMs to make the optimal option, this operator is also very important.

**Table 4:** A different ranking by altering the parameter values.

q-values	Values of score function	Ranking
q = 2	$Scr({\mathcal {N}}_{1})=1.8367$, $Scr({\mathcal {N}}_{2})=1.7825$, $Scr({\mathcal {N}}_{3})=1.1859$, $Scr({\mathcal {N}}_{4})=1.5328$, $Scr({\mathcal {N}}_{5})=1.3585$	${\mathcal {N}}_{1}>{\mathcal {N}}_{2}>{\mathcal {N}}_{4}>{\mathcal {N}}_{5}>{\mathcal {N}}_{3}$
q = 4	$Scr({\mathcal {N}}_{1})=2.2474$, $Scr({\mathcal {N}}_{2})=1.4528$, $Scr({\mathcal {N}}_{3})=1.3629$, $Scr({\mathcal {N}}_{4})=1.5717$, $Scr({\mathcal {N}}_{5})=1.4715$	${\mathcal {N}}_{1}>{\mathcal {N}}_{4}>{\mathcal {N}}_{5}>{\mathcal {N}}_{2}>{\mathcal {N}}_{3}$
q = 8	$Scr({\mathcal {N}}_{1})=2.7875$, $Scr({\mathcal {N}}_{2})=1.8494$, $Scr({\mathcal {N}}_{3})=1.6129$, $Scr({\mathcal {N}}_{4})=1.8614$, $Scr({\mathcal {N}}_{5})=1.7006$	${\mathcal {N}}_{1}>{\mathcal {N}}_{4}>{\mathcal {N}}_{2}>{\mathcal {N}}_{5}>{\mathcal {N}}_{3}$
q = 10	$Scr({\mathcal {N}}_{1})=2.9275$, $Scr({\mathcal {N}}_{2})=1.9718$, $Scr({\mathcal {N}}_{3})=1.6709$, $Scr({\mathcal {N}}_{4})=1.9597$, $Scr({\mathcal {N}}_{5})=1.7578$	${\mathcal {N}}_{1}>{\mathcal {N}}_{2}>{\mathcal {N}}_{4}>{\mathcal {N}}_{5}>{\mathcal {N}}_{3}$
q = 12	$Scr({\mathcal {N}}_{1})=3.0246$, $Scr({\mathcal {N}}_{2})=2.0629$, $Scr({\mathcal {N}}_{3})=1.7089$, $Scr({\mathcal {N}}_{4})=2.0280$, $Scr({\mathcal {N}}_{5})=1.7916$	${\mathcal {N}}_{1}>{\mathcal {N}}_{2}>{\mathcal {N}}_{4}>{\mathcal {N}}_{5}>{\mathcal {N}}_{3}$
q = 15	$Scr({\mathcal {N}}_{1})=3.1227$, $Scr({\mathcal {N}}_{2})=2.1599$, $Scr({\mathcal {N}}_{3})=1.7459$, $Scr({\mathcal {N}}_{4})=2.0976$, $Scr({\mathcal {N}}_{5})=1.8194$	${\mathcal {N}}_{1}>{\mathcal {N}}_{2}>{\mathcal {N}}_{4}>{\mathcal {N}}_{5}>{\mathcal {N}}_{3}$

### Sensitivity analysis w.r.t. weights of characteristics

In order to assess the effects of various weightings given to the qualities under consideration, sensitivity analysis with respect to attribute weights is a technique used in decision-making procedures, notably in MCDM. Multiple traits or criteria are frequently assessed in these decision-making situations, and these attributes may be given varying weights or degrees of relevance. The decision-maker’s preferences, domain expertise, or other considerations are frequently used to apply these weights. Sensitivity analysis for attribute weights is methodically changing the weights given to each attribute and seeing how those changes affect the final judgment or conclusion. This analysis’s objectives are to determine which qualities have the most influence on the choice and how sensitive the decision or result is to the weights given to each attribute. Sensitivity analysis would include changing the weights given to each criterion and examining the changes in the overall ranks of nanoparticles across sectors as a consequence. The industries might use this study to identify which factors are most important to their choice and change the weights of those factors accordingly. Overall, it is a helpful tool for analyzing decision-making issues and determining how resilient choices are to changes in the relative importance of various traits.

**Table 5:** Sensitivity analysis w.r.t. weights of characteristics.

Values of new weights	Values of score function	Ranking
$\{0.2655,0.1257,0.1897,0.1266,0.2925\}$	$Scr({\mathcal {N}}_{1})=2.8237$, $Scr({\mathcal {N}}_{2})=2.8181$, $Scr({\mathcal {N}}_{3})=1.6594$, $Scr({\mathcal {N}}_{4})=2.7591$, $Scr({\mathcal {N}}_{5})=2.5299$	${\mathcal {N}}_{1}>{\mathcal {N}}_{2}>{\mathcal {N}}_{4}>{\mathcal {N}}_{5}>{\mathcal {N}}_{3}$
$\{0.2860,0.1596,0.2651,0.1986,0.0911\}$	$Scr({\mathcal {N}}_{1})=3.1294$, $Scr({\mathcal {N}}_{2})=2.7215$, $Scr({\mathcal {N}}_{3})=1.9847$, $Scr({\mathcal {N}}_{4})=2.7021$, $Scr({\mathcal {N}}_{5})=2.3182$	${\mathcal {N}}_{1}>{\mathcal {N}}_{2}>{\mathcal {N}}_{4}>{\mathcal {N}}_{5}>{\mathcal {N}}_{3}$
$\{0.1971,0.1860,0.2569,0.2156,0.1444\}$	$Scr({\mathcal {N}}_{1})=3.0474$, $Scr({\mathcal {N}}_{2})=3.0281$, $Scr({\mathcal {N}}_{3})=1.9745$, $Scr({\mathcal {N}}_{4})=2.6090$, $Scr({\mathcal {N}}_{5})=2.2132$	${\mathcal {N}}_{1}>{\mathcal {N}}_{2}>{\mathcal {N}}_{4}>{\mathcal {N}}_{5}>{\mathcal {N}}_{3}$
$\{0.2151,0.1699,0.2598,0.1081,0.2471\}$	$Scr({\mathcal {N}}_{1})=1.9623$, $Scr({\mathcal {N}}_{2})=1.3800$, $Scr({\mathcal {N}}_{3})=0.9106$, $Scr({\mathcal {N}}_{4})=1.4170$, $Scr({\mathcal {N}}_{5})=1.3772$	${\mathcal {N}}_{1}>{\mathcal {N}}_{5}>{\mathcal {N}}_{2}>{\mathcal {N}}_{4}>{\mathcal {N}}_{3}$
$\{0.1616,0.2659,0.2830,0.1591,0.1304\}$	$Scr({\mathcal {N}}_{1})=2.0960$, $Scr({\mathcal {N}}_{2})=1.5521$, $Scr({\mathcal {N}}_{3})=1.1748$, $Scr({\mathcal {N}}_{4})=0.7717$, $Scr({\mathcal {N}}_{5})=1.4338$	${\mathcal {N}}_{1}>{\mathcal {N}}_{2}>{\mathcal {N}}_{5}>{\mathcal {N}}_{3}>{\mathcal {N}}_{4}$

By assigning the alternate weights, it is clear from Table 5 that ranking of nanoparticles is invariant and consistence as likely to Fig. 5. When employing various weight qualities, the order of choices is always ${\mathcal {N}}_{1}>{\mathcal {N}}_{2}>{\mathcal {N}}_{4}>{\mathcal {N}}_{5}>{\mathcal {N}}_{3}$ or a little modification to it.

## Comparison analysis

Comparative analysis is the procedure of evaluating two or more related objects to see how they are similar to and distinguishing among one another. People may be better able to appreciate the similarities and contrasts of many items by applying it in a variety of contexts and sectors. It can help businesses decide wisely on crucial issues. It may be put to good use when combined with scientific data. Scientific data is information that has been gathered via scientific research and have used for a certain purpose. When compared to scientific data, it demonstrates how exact and reliable the data is. Additionally, it helps scientists verify the reliability and quality of their data. Comparative studies are essential if we wish to comprehend a subject better or find solutions to important problems. These are the main goals that businesses employ comparative analysis to achieve. It encourages a detailed understanding of the opportunities related to certain processes, departments, or business units. This study also ensures that the real reasons of performance gaps are being addressed. It is commonly used since it helps to comprehend both the current and previous challenges that a firm has faced. This method offers objective, verifiable information on performance as well as recommendations for improving it.

We use the following comparison similarities to further illustrate the advantages and benefits of the suggested strategies.

### Comparison of the presented approach with the presented methodology by Zeng and Smarandache^58^

We use Zeng and Smarandache^58^ to address the aforementioned problem, with visible results in Table 6. In Table 6, we calculated the values of score by using existing m-polar-DNNSSWAA and m-polar-DNNSSWGA operators^58^ and compared the outcomes to the method suggested in this article. The rankings of each alternative have not changed significantly. For both methods, the top-ranked alternatives are the same. In our suggested approach, the weights attribute was known, making it more reasonable and adaptable. The weights attribute was known and extremely simple to determine the ranking of alternatives.

**Table 6:** Evaluation of existing operators in comparison to current operators^58^.

Different operators	Score function values	Ranking
m-polar-DNNSSWAA^58^	$Scr({\mathcal {N}}_{1})=0.4425$, $Scr({\mathcal {N}}_{2})=0.4718$, $Scr({\mathcal {N}}_{3})=0.4449$, $Scr({\mathcal {N}}_{4})=0.4556$, $Scr({\mathcal {N}}_{5})=0.4204$	${\mathcal {N}}_{2}>{\mathcal {N}}_{4}>{\mathcal {N}}_{3}>{\mathcal {N}}_{1}>{\mathcal {N}}_{5}$
m-polar-DNNSSWGA^58^	$Scr({\mathcal {N}}_{1})=0.3829$, $Scr({\mathcal {N}}_{2})=0.3568$, $Scr({\mathcal {N}}_{3})=0.3650$, $Scr({\mathcal {N}}_{4})=0.3673$, $Scr({\mathcal {N}}_{5})=0.3711$	${\mathcal {N}}_{1}>{\mathcal {N}}_{5}>{\mathcal {N}}_{4}>{\mathcal {N}}_{3}>{\mathcal {N}}_{2}$
P-q-RLNWAA (proposed operator)	$Scr({\mathcal {N}}_{1})=2.9304$, $Scr({\mathcal {N}}_{2})=3.0689$, $Scr({\mathcal {N}}_{3})=2.0507$, $Scr({\mathcal {N}}_{4})=2.8417$, $Scr({\mathcal {N}}_{5})=2.4107$	${\mathcal {N}}_{2}>{\mathcal {N}}_{1}>{\mathcal {N}}_{4}>{\mathcal {N}}_{5}>{\mathcal {N}}_{3}$
P-q-RLNWGA (proposed operator)	$Scr({\mathcal {N}}_{1})=1.8361$, $Scr({\mathcal {N}}_{2})=1.2721$, $Scr({\mathcal {N}}_{3})=1.1856$, $Scr({\mathcal {N}}_{4})=1.5326$, $Scr({\mathcal {N}}_{5})=1.3580$	${\mathcal {N}}_{1}>{\mathcal {N}}_{4}>{\mathcal {N}}_{5}>{\mathcal {N}}_{2}>{\mathcal {N}}_{3}$

### Comparison of the presented approach with the presented methodology by Awang and Abdullah^59^

Additionally, to demonstrate the suitability and validity of the approach suggested in this work, we compared with Awang and Abdullah^59^ technique. In Table 7, we solved the above-explanatory example by using of HBVNWA and HBVNWG operators and compared the proposed technique results in this paper with Awang and Abdullah technique^59^. We noted that the ranking of alternatives is almost the same. In Awang and Abdullah technique^59^, In the picture fuzzy set, there is just a quantitative portion, but in our work, we have additional information about both the quantitative and qualitative parts, which we call the linguistic parts. We observed that when are using^59^ it shows that our approach is more effective and instructive.

**Table 7:** Evaluation of existing operators in comparison to current operators^59^.

Different operators	Score function values	Ranking
HBVNWA^59^	$Scr({\mathcal {N}}_{1})=0.4204$, $Scr({\mathcal {N}}_{2})=0.4098$, $Scr({\mathcal {N}}_{3})=0.3543$, $Scr({\mathcal {N}}_{4})=0.3879$, $Scr({\mathcal {N}}_{5})=0.3776$	${\mathcal {N}}_{1}>{\mathcal {N}}_{2}>{\mathcal {N}}_{4}>{\mathcal {N}}_{5}>{\mathcal {N}}_{3}$
HBVNWG^59^	$Scr({\mathcal {N}}_{1})=0.3904$, $Scr({\mathcal {N}}_{2})=0.4100$, $Scr({\mathcal {N}}_{3})=0.3785$, $Scr({\mathcal {N}}_{4})=0.3865$, $Scr({\mathcal {N}}_{5})=0.3798$	${\mathcal {N}}_{2}>{\mathcal {N}}_{1}>{\mathcal {N}}_{4}>{\mathcal {N}}_{5}>{\mathcal {N}}_{3}$
P-q-RLNWAA (proposed operator)	$Scr({\mathcal {N}}_{1})=2.9304$, $Scr({\mathcal {N}}_{2})=3.0689$, $Scr({\mathcal {N}}_{3})=2.0507$, $Scr({\mathcal {N}}_{4})=2.8417$, $Scr({\mathcal {N}}_{5})=2.4107$	${\mathcal {N}}_{2}>{\mathcal {N}}_{1}>{\mathcal {N}}_{4}>{\mathcal {N}}_{5}>{\mathcal {N}}_{3}$
P-q-RLNWGA (proposed operator)	$Scr({\mathcal {N}}_{1})=1.8361$, $Scr({\mathcal {N}}_{2})=1.2721$, $Scr({\mathcal {N}}_{3})=1.1856$, $Scr({\mathcal {N}}_{4})=1.5326$, $Scr({\mathcal {N}}_{5})=1.3580$	${\mathcal {N}}_{1}>{\mathcal {N}}_{4}>{\mathcal {N}}_{5}>{\mathcal {N}}_{2}>{\mathcal {N}}_{3}$

The analysis presented above demonstrates the effectiveness of our proposed strategies for solving decision-making (DM) issues, particularly for multiple attribute decision-making (MADM). Compared to other approaches, our methods offer greater flexibility and rationality for addressing MADM challenges. These advantages are largely attributed to the use of probabilistic q-rung linguistic neutrosophic (P-q-RLNS), which allow DMs to show their opinions more freely while minimizing data loss. P-q-RLNS is relevant and sufficient for reflecting assessments of different possibilities since our method also takes into account the quantitative presumptions that decision-makers frequently make while making subjective judgments. Additionally, P-q-RLNSWAA or P-q-RLNSWGA, which alert users to the links between various traits or criteria, are the foundation of our MADM approach. As a result, decision-makers now have a new tool at their disposal for conveying their evaluations, increasing the effectiveness of our technique in simulating real-world MADM difficulties. Our strategy is more comprehensive, robust, and adaptable than other approaches, which helps it be a successful remedy for dealing with MADM problems.

We have provided a more detailed explanation of the advantages of our proposed approach, especially in comparison to existing studies. We highlighted how our method outperforms others by utilizing a richer set of data. The expanded discussion and additional results in the latest version underscore the strengths of our approach, making the comparison more effective. Furthermore, we have expanded the experimental setup to provide a more comprehensive understanding of the methodology and to showcase the robustness of our proposed method.

**Table 8:** Comparison of the study proposal with current relevant structures.

Name	Year	Structure	Probabilistic	Ling.	Mem.	Ind.	N-Mem.
Zadeh^60^	1965	Fuzzy set	$\times$	$\times$	$\checkmark$	$\times$	$\times$
Atanassov^61^	1986	Intuitionistic fuzzy set	$\times$	$\times$	$\checkmark$	$\times$	$\checkmark$
Yager et al.^62^	2013	Pythagorean fuzzy set	$\times$	$\times$	$\checkmark$	$\times$	$\checkmark$
Yager^63^	2016	Q-rung orthopair fuzzy set	$\times$	$\times$	$\checkmark$	$\times$	$\checkmark$
Smarandache et al.^64^	2005	Neutrosophic Set	$\times$	$\times$	$\checkmark$	$\checkmark$	$\checkmark$
Bhowmki et al.^65^	2009	Intuitionistic neutrosophic set	$\times$	$\times$	$\checkmark$	$\checkmark$	$\checkmark$
Jansi et al.^66^	2019	Pythagorean neutrosophic set	$\times$	$\times$	$\checkmark$	$\checkmark$	$\checkmark$
Ali et al.^54^	2022	Q-rung linguistic Picture Fuzzy Set	$\times$	$\checkmark$	$\checkmark$	$\checkmark$	$\checkmark$
Proposed technique	2023	Probabilistic Q-rung linguistic neutrosophic set	$\checkmark$	$\checkmark$	$\checkmark$	$\checkmark$	$\checkmark$

In Ali et al.^54^ structure (GQRPFL) has a limitation. It deals when the existing model exist in the form of q-rung liguistic picture fuzzy set with the condition $0\le \Theta _{B_{2}}(\ddot{m})+\Psi _{B_{2}}(\ddot{m})+\Pi _{B_{2}}(\ddot{m})\le 1$. But in our proposed structure (P-QRLNS) deals when the $0\le \Theta _{B_{2}}(\ddot{m})+\Psi _{B_{2}}(\ddot{m})+\Pi _{B_{2}}(\ddot{m})\le 3$, then we generalized it by using possibility term and increasing the power of Membership, indeterminacy and Non-Membership upto “q” to adjust the value in closed intervals 0 and 3 as shown in Table 8.

## Conclusions and future initiatives

In conclusion, our proposed algorithmic framework advances the assessment of nanoparticles for industrial use. This innovative approach navigates complexity and uncertainty in nanofluid evaluation, enabling precise decision-making. By systematically considering factors and uncertainties, it enhances nanofluid selection in industries. The aggregation operator enables robust nanoparticle comparisons, providing a valuable tool for informed decision-making and contributing to efficiency and sustainability in industrial processes. To summarize, our study contributes by integrating probabilistic q-rung orthopair linguistic neutrosophic quantification, offering a nuanced understanding of nanofluid assessment, and an effective aggregation operator for streamlined decision-making. Findings highlight the framework’s efficacy, empowering industry professionals for judicious nanoparticle selections. Further validation and exploration across diverse industrial contexts are recommended, underscoring the framework’s potential impact. Overall, this research optimizes industrial processes through enhanced decision support in nanoparticle selection.

The future directions for a comprehensive approach to nanoparticles ranking which used in industries assessment using probabilistic q-rung linguistic neutrosophic weighted aggregation operators involve probabilistic q-rung linguistic orthopair interval-valued neutrosophic fuzzy soft set, probabilistic q-rung linguistic orthopair bi-polar neutrosophic fuzzy soft set, probabilistic q-rung linguistic orthopair m-polar neutrosophic fuzzy soft set, probabilistic q-rung linguistic orthopair cubic neutrosophic fuzzy soft set and probabilistic q-rung linguistic orthopair neutrosophic fuzzy soft set etc.

### Ethical approval

This study does not involve the use of human subjects or animals, therefore ethical approval is not required. The research relies exclusively on publicly available data, and no personally identifiable information is being collected or analyzed. All procedures and methodologies strictly adhere to established ethical guidelines and regulations.

### Supplementary Information

Supplementary Information.

## Data Availability

The datasets used and analysed during the current study are available from the corresponding author on reasonable request.

## References

[1] Munson, B. R., Okiishi, T. H., Huebsch, W. W. & Rothmayer, A. P. *Fluid Mechanics* (Wiley Singapore, 2013).

[2] Choi, S. U. & Eastman, J. A. “Enhancing thermal conductivity of fluids with nanoparticles,” tech. rep., Argonne National Lab.(ANL), Argonne, IL (United States), (1995).

[3] Xuan, Y. & Li, Q. Heat transfer enhancement of nanofluids. *Int. J. Heat Fluid Flow* **21**(1), 58–64 (2000). 10.1016/S0142-727X(99)00067-3

[4] Wang, X.-Q. & Mujumdar, A. S. A review on nanofluids-part II: Experiments and applications. *Braz. J. Chem. Eng.* **25**, 631–648 (2008). 10.1590/S0104-66322008000400002

[5] Heris, S. Z., Esfahany, M. N. & Etemad, S. G. Experimental investigation of convective heat transfer of al2o3/water nanofluid in circular tube. *Int. J. Heat Fluid Flow* **28**(2), 203–210 (2007). 10.1016/j.ijheatfluidflow.2006.05.001

[6] Prasad, A. R., Singh, S. & Nagar, H. A review on nanofluids: Properties and applications. *Int. J. Adv. Res. Innov. Ideas Educ.* **3**(3), 3185–3209 (2017).

[7] Bashirnezhad, K. *et al.* Viscosity of nanofluids: A review of recent experimental studies. *Int. Commun. Heat Mass Transf* **73**, 114–123 (2016). 10.1016/j.icheatmasstransfer.2016.02.005

[8] Shahid, A., Zhou, Z., Hassan, M. & Bhatti, M. M. Computational study of magnetized blood flow in the presence of gyrotactic microorganisms propelled through a permeable capillary in a stretching motion. *Int. J. Multiscale Comput. Eng.* **16**(5), 409–426 (2018).

[9] Clifford, A. A. & Williams, J. R. *Introduction to Supercritical Fluids and Their Applications* (Springer, 2000).

[10] Chamsa-Ard, W., Brundavanam, S., Fung, C. C., Fawcett, D. & Poinern, G. Nanofluid types, their synthesis, properties and incorporation in direct solar thermal collectors: A review. *Nanomaterials* **7**(6), 131 (2017). 28561802 10.3390/nano7060131 PMC5485778

[11] Klir, G. & Yuan, B. *Fuzzy Sets and Fuzzy Logic* Vol. 4 (Prentice Hall, 1995).

[12] Edwards, W. The theory of decision making. *Psychol. Bull.* **51**(4), 380 (1954). 13177802 10.1037/h0053870

[13] Zimmermann, H.-J. *Fuzzy Set Theory-and Its Applications* (Springer Science & Business Media, 2011).

[14] De, S. K., Biswas, R. & Roy, A. R. An application of intuitionistic fuzzy sets in medical diagnosis. *Fuzzy Sets Syst.* **117**(2), 209–213 (2001). 10.1016/S0165-0114(98)00235-8

[15] Torra, V. Hesitant fuzzy sets. *Int. J. Intell. Syst.* **25**(6), 529–539 (2010).

[16] Zadeh, L. A. The concept of a linguistic variable and its application to approximate reasoning-i. *Inf. Sci.* **8**(3), 199–249 (1975). 10.1016/0020-0255(75)90036-5

[17] Maiers, J. & Sherif, Y. S. Applications of fuzzy set theory. *IEEE Trans. Syst. Man Cybern.* **1**, 175–189 (1985). 10.1109/TSMC.1985.6313408

[18] Khan, M. J., Kumam, P. & Shutaywi, M. Knowledge measure for the q-rung orthopair fuzzy sets. *Int. J. Intell. Syst.* **36**(2), 628–655 (2021). 10.1002/int.22313

[19] Ejegwa, P. A. New q-rung orthopair fuzzy distance-similarity operators with applications in investment analysis, pattern recognition, clustering analysis, and selection of robot for smart manufacturing. *Soft Comput.* 1–20. 10.1007/s00500-023-08799-1 (2023).

[20] Ejegwa, P. A. & Davvaz, B. An improved composite relation and its application in deciding patients medical status based on a q-rung orthopair fuzzy information. *Comput. Appl. Math.* **41**(7), 303 (2022). 10.1007/s40314-022-02005-y

[21] Ejegwa, P. A. & Sarkar, A. Novel correlation measure for generalized orthopair fuzzy sets and its decision-making applications. In *Operations Research Forum*, vol. 4, 32 (Springer, 2023).

[22] Ejegwa, P. A. Decision-making on patients’ medical status based on a q-rung orthopair fuzzy max-min-max composite relation. In *q-Rung Orthopair Fuzzy Sets: Theory and Applications*, 47–66 (Springer, 2022).

[23] Joshi, B. P., Singh, A., Bhatt, P. K. & Vaisla, K. S. Interval valued q-rung orthopair fuzzy sets and their properties. *J. Intell. Fuzzy Syst.* **35**(5), 5225–5230 (2018). 10.3233/JIFS-169806

[24] Salama, A. & Smarandache, F. Neutrosophic crisp set theory. *Neutrosophic Sets Syst.* **5**, 27–35 (2014).

[25] Saeed, M., Wahab, A., Ali, J. & Bonyah, E. A robust algorithmic framework for the evaluation of international cricket batters in odi format based on q-rung linguistic neutrosophic quantification. *Heliyon*. **9**(11), 1–20. 10.1016/j.heliyon.2023.e21429 (2023). 10.1016/j.heliyon.2023.e21429 PMC10628702 37942171

[26] El-Hefenawy, N., Metwally, M. A., Ahmed, Z. M. & El-Henawy, I. M. A review on the applications of neutrosophic sets. *J. Comput. Theor. Nanosci.* **13**(1), 936–944 (2016). 10.1166/jctn.2016.4896

[27] Bhaumik, A., Roy, S. K. & Weber, G. W. Multi-objective linguistic-neutrosophic matrix game and its applications to tourism management. *J. Dyn. Games* **8**(2), 101–118 (2021). 10.3934/jdg.2020031

[28] Das, S., Roy, B. K., Kar, M. B., Kar, S. & Pamučar, D. Neutrosophic fuzzy set and its application in decision making. *J. Ambient. Intell. Humaniz. Comput.* **11**, 5017–5029 (2020). 10.1007/s12652-020-01808-3

[29] Xing, Y., Zhang, R., Zhu, X. & Bai, K. q-rung orthopair fuzzy uncertain linguistic choquet integral operators and their application to multi-attribute decision making. *J. Intell. Fuzzy Syst.* **37**(1), 1123–1139 (2019). 10.3233/JIFS-182581

[30] Kuo, T. Interval multiplicative pairwise comparison matrix: Consistency, indeterminacy and normality. *Inf. Sci.* **517**, 244–253 (2020). 10.1016/j.ins.2019.12.066

[31] Xu, Y., Chen, L., Rodríguez, R. M., Herrera, F. & Wang, H. Deriving the priority weights from incomplete hesitant fuzzy preference relations in group decision making. *Knowl.-Based Syst.* **99**, 71–78 (2016). 10.1016/j.knosys.2016.01.047

[32] Kamacı, H. Linguistic single-valued neutrosophic soft sets with applications in game theory. *Int. J. Intell. Syst.* **36**(8), 3917–3960 (2021). 10.1002/int.22445

[33] Saeed, M., Wahab, A., Ali, M., Ali, J. & Bonyah, E. An innovative approach to passport quality assessment based on the possibility q-rung ortho-pair fuzzy hypersoft set. *Heliyon*. **9**(9), 1–18. 10.1016/j.heliyon.2023.e19379 (2023). 10.1016/j.heliyon.2023.e19379 PMC10480657 37681123

[34] Pennington, N. & Hastie, R. Evidence evaluation in complex decision making. *J. Pers. Soc. Psychol.* **51**(2), 242 (1986). 10.1037/0022-3514.51.2.242

[35] Chai, J., Liu, J. N. & Ngai, E. W. Application of decision-making techniques in supplier selection: A systematic review of literature. *Expert Syst. Appl.* **40**(10), 3872–3885 (2013). 10.1016/j.eswa.2012.12.040

[36] Herrera, F. & Herrera-Viedma, E. Aggregation operators for linguistic weighted information. *IEEE Trans. Syst. Man Cybern. Part A Syst. Hum.* **27**(5), 646–656 (1997). 10.1109/3468.618263

[37] Senapati, T. & Yager, R. R. Fermatean fuzzy weighted averaging/geometric operators and its application in multi-criteria decision-making methods. *Eng. Appl. Artif. Intell.* **85**, 112–121 (2019). 10.1016/j.engappai.2019.05.012

[38] Zadeh, L. A., Klir, G. J. & Yuan, B. *Fuzzy Sets, Fuzzy Logic, and Fuzzy Systems: Selected Papers* Vol. 6 (World Scientific, 1996).

[39] Zadeh, L. A. Fuzzy sets and information granularity. In *Fuzzy Sets, Fuzzy Logic, and Fuzzy Systems: Selected Papers*, 433–448 (1979).

[40] Mizumoto, M. & Tanaka, K. Some properties of fuzzy sets of type 2. *Inf. Control* **31**(4), 312–340 (1976). 10.1016/S0019-9958(76)80011-3

[41] Ali, M. I. Another view on q-rung orthopair fuzzy sets. *Int. J. Intell. Syst.* **33**(11), 2139–2153 (2018). 10.1002/int.22007

[42] Oh, H., Kim, H., Kim, H. & Kim, C. A method for improving the multiplicative inconsistency based on indeterminacy of an intuitionistic fuzzy preference relation. *Inf. Sci.* **602**, 1–12 (2022). 10.1016/j.ins.2022.03.086

[43] Alblowi, S., Salama, A. & Eisa, M. *New concepts of neutrosophic sets*. Infinite Study, (2014).

[44] Mallick, R. & Pramanik, S. Pentapartitioned neutrosophic set and its properties, vol. 36. Infinite Study, (2020).

[45] Khalil, A. M., Cao, D., Azzam, A., Smarandache, F. & Alharbi, W. R. Combination of the single-valued neutrosophic fuzzy set and the soft set with applications in decision-making. *Symmetry* **12**(8), 1361 (2020). 10.3390/sym12081361

[46] Smarandache, F. Neutrosophic set is a generalization of intuitionistic fuzzy set, inconsistent intuitionistic fuzzy set (picture fuzzy set, ternary fuzzy set), pythagorean fuzzy set, spherical fuzzy set, and q-rung orthopair fuzzy set, while neutrosophication is a generalization of regret theory, grey system theory, and three-ways decision (revisited). *J. New Theory* **29**, 1–31 (2019).

[47] Drossos, C. A. Generalized t-norm structures. *Fuzzy Sets Syst.* **104**(1), 53–59 (1999). 10.1016/S0165-0114(98)00258-9

[48] Murofushi, T. & Sugeno, M. Fuzzy t-conorm integral with respect to fuzzy measures: generalization of sugeno integral and choquet integral. *Fuzzy Sets Syst.* **42**(1), 57–71 (1991). 10.1016/0165-0114(91)90089-9

[49] Jenei, S. On Archimedean triangular norms. *Fuzzy Sets Syst.* **99**(2), 179–186 (1998). 10.1016/S0165-0114(97)00021-3

[50] Li, Z., Zhao, C. & Zheng, P. Operations on hesitant linguistic terms sets induced by Archimedean triangular norms and conorms. *Int. J. Comput. Intell. Syst.* **11**(1), 514 (2018). 10.2991/ijcis.11.1.38

[51] Kleijnen, J. P. & Rubinstein, R. Y. Optimization and sensitivity analysis of computer simulation models by the score function method. *Eur. J. Oper. Res.* **88**(3), 413–427 (1996). 10.1016/0377-2217(95)00107-7

[52] Kliegl, R., Maayr, U. & Krampe, R. T. Time-accuracy functions for determining process and person differences: An application to cognitive aging. *Cogn. Psychol.* **26**(2), 134–164 (1994). 8205771 10.1006/cogp.1994.1005

[53] Kokoç, M. & Ersöz, S. New score and accuracy function for IVIF sets and their applications to AHP for MCGDM. *Cybern. Syst.* **53**(3), 257–281 (2022). 10.1080/01969722.2021.1949519

[54] Ali, J., Naeem, M. & Mahmood, W. Generalized q-rung picture linguistic aggregation operators and their application in decision making. *J. Intell. Fuzzy Syst.* 1–25 (2023).

[55] Keikha, A. Archimedean t-norm and t-conorm-based aggregation operators of HFNs, with the approach of improving education. *Int. J. Fuzzy Syst.* **24**(1), 310–321 (2022). 10.1007/s40815-021-01137-3

[56] Liu, P. The aggregation operators based on Archimedean t-conorm and t-norm for single-valued neutrosophic numbers and their application to decision making. *Int. J. Fuzzy Syst.* **18**(5), 849–863 (2016). 10.1007/s40815-016-0195-8

[57] Chatterjee, A., Mukherjee, S. & Kar, S. A rough approximation of fuzzy soft set-based decision-making approach in supplier selection problem. *Fuzzy Inf. Eng.* **10**(2), 178–195 (2018). 10.1080/16168658.2018.1517973

[58] Zeng, S., Ali, S., Mahmood, M. K., Smarandache, F. & Ahmad, D. Decision-making problems under the environment of m-polar diophantine neutrosophic n-soft set. *Comput. Model. Eng. Sci.* **130**, 581–606 (2022).

[59] Awang, A., Ali, M. & Abdullah, L. Hesitant bipolar-valued neutrosophic set: Formulation, theory and application. *IEEE Access* **7**, 176099–176114 (2019). 10.1109/ACCESS.2019.2946985

[60] Zadeh, L. Fuzzy sets. *Inform. Control* **8**, 338–353 (1965). 10.1016/S0019-9958(65)90241-X

[61] Atanassov, K. T. & Atanassov, K. T. *Intuitionistic Fuzzy Sets* (Springer, 1999).

[62] Yager, R. R. Pythagorean fuzzy subsets. In *2013 Joint IFSA World Congress and NAFIPS Annual Meeting (IFSA/NAFIPS)*, 57–61 (IEEE, 2013).

[63] Yager, R. R. Generalized orthopair fuzzy sets. *IEEE Trans. Fuzzy Syst.* **25**(5), 1222–1230 (2016). 10.1109/TFUZZ.2016.2604005

[64] Smarandache, F. Neutrosophic set—a generalization of the intuitionistic fuzzy set. *Int. J. Pure Appl. Math.* **24**(3), 287 (2005).

[65] Bhowmik, M. & Pal, M. *Intuitionistic neutrosophic set*. Infinite Study, (2009).

[66] Jansi, R., Mohana, K. & Smarandache, F. *Correlation measure for pythagorean neutrosophic sets with t and f as dependent neutrosophic components*. Infinite Study, (2019).

